# Sodium bicarbonate cotransporter NBCe2 gene variants increase sodium and bicarbonate transport in human renal proximal tubule cells

**DOI:** 10.1371/journal.pone.0189464

**Published:** 2018-04-11

**Authors:** John J. Gildea, Peng Xu, Brandon A. Kemp, Julia M. Carlson, Hanh T. Tran, Dora Bigler Wang, Christophe J. Langouët-Astrié, Helen E. McGrath, Robert M. Carey, Pedro A. Jose, Robin A. Felder

**Affiliations:** 1 The University of Virginia Department of Pathology, Charlottesville, VA, United States of America; 2 The University of Virginia Department of Medicine, Charlottesville, VA, United States of America; 3 The George Washington University School of Medicine & Health Sciences, Department of Medicine, Division of Renal Disease and Hypertension and Department of Pharmacology and Physiology, Washington, DC, United States of America; University of Fribourg, SWITZERLAND

## Abstract

**Rationale:**

Salt sensitivity of blood pressure affects >30% of the hypertensive and >15% of the normotensive population. Variants of the electrogenic sodium bicarbonate cotransporter NBCe2 gene, *SLC4A5*, are associated with increased blood pressure in several ethnic groups. *SLC4A5* variants are also highly associated with salt sensitivity, independent of hypertension. However, little is known about how NBCe2 contributes to salt sensitivity, although NBCe2 regulates renal tubular sodium bicarbonate transport. We hypothesized that *SLC4A5* rs10177833 and rs7571842 increase NBCe2 expression and human renal proximal tubule cell (hRPTC) sodium transport and may be a cause of salt sensitivity of blood pressure.

**Objective:**

To characterize the hRPTC ion transport of wild-type (WT) and homozygous variants (HV) of *SLC4A5*.

**Methods and results:**

The expressions of NBCe2 mRNA and protein were not different between hRPTCs carrying WT or HV *SLC4A5* before or after dopaminergic or angiotensin (II and III) stimulation. However, luminal to basolateral sodium transport, NHE3 protein, and Cl^-^/HCO_3_^-^ exchanger activity in hRPTCs were higher in HV than WT *SLC4A5*. Increasing intracellular sodium enhanced the apical location of NBCe2 in HV hRPTCs (4.24±0.35% to 11.06±1.72% (P<0.05, N = 3, 2-way ANOVA, Holm-Sidak test)) as determined by Total Internal Reflection Fluorescence Microscopy (TIRFM). In hRPTCs isolated from kidney tissue, increasing intracellular sodium enhanced bicarbonate-dependent pH recovery rate and increased NBCe2 mRNA and protein expressions to a greater extent in HV than WT *SLC4A5* (+38.00±6.23% vs HV normal salt (P<0.01, N = 4, 2-way ANOVA, Holm-Sidak test)). In hRPTCs isolated from freshly voided urine, bicarbonate-dependent pH recovery was also faster in those from salt-sensitive and carriers of HV *SLC4A5* than from salt-resistant and carriers of WT *SLC4A5*. The faster NBCe2-specific bicarbonate-dependent pH recovery rate in HV *SCL4A5* was normalized by *SLC4A5*- but not *SLC4A4*-shRNA. The binding of purified hepatocyte nuclear factor type 4A (HNF4A) to DNA was increased in hRPTCs carrying HV *SLC4A5* rs7571842 but not rs10177833. The faster NBCe2-specific bicarbonate-dependent pH recovery rate in HV *SCL4A5* was abolished by HNF4A antagonists.

**Conclusion:**

NBCe2 activity is stimulated by an increase in intracellular sodium and is hyper-responsive in hRPTCs carrying HV *SLC4A5* rs7571842 through an aberrant HNF4A-mediated mechanism.

## Introduction

Hypertension and salt sensitivity of blood pressure (BP) have genetic and environmental components. Salt sensitivity is observed in 30–60% of hypertensive and 15–26% of normotensive adults. Salt sensitivity, *per se*, independent of blood pressure has similar cardiovascular morbidity and mortality to hypertension [1], yet is difficult to diagnose[2]. Over the past decade, several candidate genes involved in the regulation of sodium homeostasis have been associated with salt sensitivity. Two studies linked loci in chromosome 2 to a higher resting BP in Nigerians, as well as both African- and Caucasian-Americans[3, 4]. The locus was mapped to chromosome 2p14-2p13.1 [5, 6] containing the *SLC4A5* gene, encoding the electrogenic sodium-bicarbonate cotransporter 4 (NBCe2, formerly known as NBC4)[7]. Barkley *et al* identified *SLC4A5* as the only gene in chromosome 2 that was significantly associated with hypertension within a pool of 82 single nucleotide polymorphisms (SNPs) within eight genes of interest[8]. Several SNPs within *SLC4A5*, including rs10177833 and rs7571842, had been associated with higher resting blood and pulse pressures in Caucasian- and African-Americans and Chinese[9–13]. Recently, Carey et al reported that *SLC4A5* rs10177833 and rs7571842 were highly associated with salt sensitivity, independent of hypertension, in two independent cohorts[14]. However, little is known about the normal cellular expression and function of NBCe2 in the kidney and if genetic variants of *SLC4A5* contribute to renal pathophysiology[15].

The rat kidney expresses NBCe2 to a greater extent in the medullary thick ascending limb (mTAL) and cortical thick ascending limb (cTAL) and to a lesser extent in the proximal straight tubule and cortical collecting duct (CCD)[16]. Xu et al hypothesized that NBCe2 should be located at the basolateral membrane of the mTAL and cTAL[16] because there was no measurable sodium-dependent bicarbonate transport activity in the lumens of these nephron segments. However, those studies were performed under normal but not high sodium intake[16]. We have previously reported that in kidney slices incubated with 120 mM NaCl, NBCe2 was localized particularly in the subapical membrane and in highly compartmentalized perinuclear Golgi bodies [17]. Increasing intracellular sodium by increasing extracellular sodium concentration (170 mM NaCl, in the short-term (30 min), increased the luminal expression of NBCe2, observed by confocal microscopy [17]. Furthermore, electron microscopy revealed that NBCe2 was present in a subapical compartment in the hRPTC under 120 mM NaCl conditions and migrated into the microvilli under high sodium (170 mM) conditions[17]. However, in those studies, we did not perform longer term experiments that examined transcriptional regulation of NBCe2 via its gene *SLC4A5*.

A related sodium-bicarbonate transporter, NBCe1, is located in human RPT,[18] TAL,[19] and CD[20]. NBCe1 mediates the transport of sodium and bicarbonate from inside the cell across the basolateral membrane to the basolateral space; mutations in this gene cause severe metabolic acidosis[18, 21]. NaCl or NaHCO_3_ loading in normotensive rats decreases the renal expression of both NBCe1 and sodium hydrogen exchanger 3 (NHE3)[22]. NBCe1 expression is also decreased in hRPTCs isolated from spontaneously hypertensive rats (SHR) compared with hRPTCs from normotensive Wistar-Kyoto rats[23]. However, in salt-sensitive (SS) humans, sodium transport in the RPT is increased and not decreased by an increase in sodium intake[24–26]. The increased sodium transport in the RPT of SHRs may be related to increased angiotensin II activation or an impaired dopaminergic-inhibition of renal sodium transport due to NHE3, Na^+^,K^+^/ATPase, and sodium bicarbonate [27–29] or changes in abundance of NBCe1[30]. The fact that sodium transport through NHE3 and at least 60% of bicarbonate and water transport are linked in the RPT suggest that luminal NHE3 and sodium bicarbonate transport may be functionally associated [31]. RPT bicarbonate transport is increased in hypertension [32–34]. We hypothesized that the sodium bicarbonate symporter involved in hypertension is NBCe2 because NBCe1 function in the RPT is decreased in a rat model of hypertension. [(23)] Furthermore, we hypothesized that gene variants in NBCe2 would increase sodium bicarbonate transport contributing to salt sensitivity and eventually hypertension.

Dopamine (via D1-like receptors) decreases [2, 25, 28, 29, 35–38], while angiotensin II (Ang II, via angiotensin type 1 receptor) increases [2, 24–26, 28, 30, 35, 39] sodium and bicarbonate transport in the RPT, especially under conditions of sodium loading and sodium depletion, respectively. We therefore hypothesized that gene variants of SLC4A5 would cause it to be differentially regulated by the dopaminergic or renin angiotensin systems.

Transcriptional gene regulation is important in normal and disease states [40]. Hepatocyte nuclear factor 4A (HNF4A) is one of the members of the nuclear receptor family of ligand-dependent transcription factors. HNF4A plays a key role in RPT development [41, 42], electrolyte balance in osmotically-challenged killifish,[43] and survival of severely salt challenged *C*. *elegans*[44]. HNF4A expression is increased in Dahl SS rats and is implicated in the expression of a large number of genes found in hypertension-related quantitative trait loci studies in humans and rats [45]. HNF4A has also been shown to regulate multiple components of the renin-angiotensin system (RAS) [46]. HNF4A also regulates G protein-coupled receptor kinase type 4 (aka GPRK2L) expression [47] which in turn regulates the activities of the natriuretic renal dopaminergic system and antinatriuretic RAS, aberrant regulation of which may cause salt sensitivity [35].

Thus, the current study examined the regulation and function of NBCe2 and NBCe1, using hRPTCs expressing wild-type (WT) or homozygous variants (HV) of *SLC4A5* (rs1017783 and rs757184). We tested the hypothesis that these *SLC4A5* SNPs that are associated with salt sensitivity of BP would increase the expression and activity of the gene product, NBCe2, resulting in an increase in sodium transport in hRPTCs. We further tested the hypothesis that increased expression and activity of NBCe2 caused by the presence of *SLC4A5* SNPs results from an aberrant interaction between HV *SLC4A5* with the transcriptional regulator HNF4A.

## Materials and methods

The human tissues used in our studies were obtained in accordance with a University of Virginia Institutional Review Board-approved protocol that adheres to the Declaration of Helsinki and the most recent version of the USA Code of Federal Regulations Title 45, Part 46.

### hRPTC cultures and drug treatments

#### A. primary and immortalized hRPTC culture

Ten different hRPTC lines were isolated from ten different kidney specimens from ten different subjects, as previously described[17, 36, 48, 49]. These cell lines have been extensively characterized using hRPTC-specific markers [36, 49]. Primary (pre-immortalized) and immortalized hRPTC were used. All cell lines were DNA fingerprinted to validate their origin and continuity. Four of the cell lines obtained from four different subjects were genotyped by sequencing as having no rs10177833 and rs7571842 *SLC4A5* SNPs; these were designated as wild-type (WT). The other six hRPTC lines were obtained from six other subjects expressing SNPs at both rs10177833 and rs7571842 in the *SLC4A5* gene; these were designated homozygous variant (HV).

The growth conditions for renal tissue-derived hRPTCs and urine-derived hRPTCs and drugs to block transporters, receptors, and second messengers are as follows.

The hRPTCs were grown at 37°C in full humidity with 95% air and 5% CO_2_. The cells were fed DMEM-F12 media (Invitrogen) supplemented with 2% fetal calf serum (FCS), 5 μg/mL plasmocin (InvivoGen), 10 ng/mL epidermal growth factor (Sigma), 36 ng/mL dexamethasone (Sigma), 2 ng/mL triiodothyronine (Sigma), 1x insulin/transferrin/selenium (Invitrogen), 1x penicillin/streptomycin (Invitrogen), and 0.2 mg/mL G418 sulfate (EMD Chemicals).

#### Exfoliated hRPTCs obtained from freshly voided urine

hRPTCs isolated from freshly voided urine from three SS subjects from our clinical study who carried *SLC4A5* variants were compared with three salt-resistant (SR) subjects who carried WT *SLC4A5*. hRPTCs were isolated by magnetic immuno-affinity purification, but using CD-15 instead of CD-13 (Miltenyi). The collected cells were plated onto poly-l-lysine (Sigma, 150-300k MW)-coated glass bottom 96-well plates.

The hRPTCs were plated onto 6-well plates for experiments measuring mRNA and protein expression. When the cells reached approximately 70% confluence, they were serum-starved for 24 h before sodium or drug treatment; each experiment was performed in triplicate in all ten cell lines from ten different individuals. Intracellular sodium was elevated by increasing the sodium concentration in the incubation buffer or by adding the ionophore monensin, as previously described in various model systems[17, 50–56]. All drugs tested are listed below, but the only one that elicited changes in NBCe2 expression and function was the ionophore monensin (or 170 mmol/L, extracellular NaCl). The concentrations used (1–10 μmol/L) were based on monensin titration and intracellular sodium calibration [57].

#### Drug treatments

The response to dopaminergic stimulation (24 h) of hRPTCs was studied using the D_1_-like (D_1_R/D_5_R) dopamine receptor agonists fenoldopam (FEN, fenoldopam mesylate, Corlopam, Hospira, Inc, 1 μmol/L) and SKF38393 (Sigma, 10 μmol/L), and the D_1_R/D_5_R antagonist LE300 (Tocris, 10 μmol/L)[36, 37].

Either an increase in extracellular sodium or the addition of the ionophore monensin [50–56] (1–10 μmol/L) was used to probe the effect of increased intracellular sodium on NBCe2 expression and activity. Monensin was used at several time points, from 1 to 24 h.

The response to angiotensin II (Ang II) and des-aspartyl Ang III stimulation (24 h) was tested in hRPTCs using 10 nmol/L Ang II (Sigma) and 10 nmol/L Ang III (Sigma). EC-33 (3-amino-4-thio-butyl sulfonate, Ki = 0.29 μM), and PC-18 (2-amino, 4-methylsulfonyl-butane-thiol, Ki = 8.0 nM) were used to block the conversion of Ang II to Ang III by aminopeptidase A and from Ang III to Ang IV by aminopeptidase N, respectively. Both EC-33 and PC-18 were generous gifts from Drs. Fournie-Zaluski and Roques (Universite Rene Descartes, Sciences Pharmaceutiques et Biologiques). 5-(N-ethyl-N-isopropyl-amiloride, (EIPA, Sigma, 10 μmol/L) was used to inhibit NHE3 activity^1^. 2,2'-(1,2-ethenediyl)bis[5-isothiocyanato-benzenesulfonic acid] (DIDS, Cayman Chemical, 500 μM) was used to inhibit the activity of anion exchangers, including NBCe1 and NBCe2 (17, 23). 1-[(2-chloro-5-nitrophenyl)sulfonyl]-2-methyl-1H-benzimidazole, BIM5078, and 2-methyl-1-[(2-methyl-5-nitrophenyl)sulfonyl]-1H-benzimidazole, BI6015 (Cayman Chemical, both at 10 μmol/L) were used to inhibit HNF4A activity[58].

### Localization of proteins of interest in cultured immortalized hRPTCs using confocal microscopy

The hRPTCs were prepared for immunofluorescence confocal microscopy after treatment with 120 to 170 mmol/L sodium chloride, as well as monensin (10 μmol/L) which is used by many investigators to increase intracellular sodium [17, 50–57]. Monensin, at this concentration, has been reported to increase intracellular sodium by about 20 mmol/L in opossum kidney cells [57].

#### Preparation of the hRPTCs for confocal microscopy

hRPTCs were grown on collagen IV-coated glass-bottom 96-well plates to 50% confluence and serum-starved overnight. They were incubated with monensin (1 or 10 μmol/L) or vehicle for 24 h and washed twice in PBS. They were then fixed for 5 min in PBS containing 4% paraformaldehyde and 1% Triton-X 100 and washed three times for 5 min with TBS. Immunofluorescence staining was performed as previously reported[49]. The fixed cells were blocked in Odyssey Blocking Buffer (LI-COR Biosciences) for 1 h. Primary antibodies used were polyclonal NBCe2 (1:250 dilution for Sigma HPA036621 and 1:400 dilution for Santa Cruz sc-168713) and mouse monoclonal NBCe1 (1:500 dilution, Sigma WH0008671M1). All cells were incubated for 1 h with gentle rocking at room temperature (RT), followed by washing (three times, 5 min/wash) in PBS-T (PBS plus 0.02% Tween-20). The cells were incubated in Odyssey Blocking Buffer (LI-COR Biosciences) with Alexa 488- (NBCe2) and Alexa 594- (NBCe1) conjugated secondary antibodies (2 μg/mL, Invitrogen) for 1 h at RT. The cells were washed three times in PBS-T and imaged using an Olympus IX81 automated multi-well spinning disk confocal microscope. CD-13-PE antibody (1:50, BD Biosciences 347837) was used to identify RPTCs. Hoechst 33342 (1:2000, Invitrogen 735969) was used to stain nuclei.

#### Confocal microscopy

The confocal microscope is an IX81, 6D (linear encoded X, and Y axis, piezo Z axis, time, wavelength, positions) spinning disk confocal with both mercury and xenon light sources and Semrock hard-coated filters in a Sedat configuration. Images were acquired using a 100x UPlanSApo oil immersion objective with NA 1.4 for cell imaging. The microscope is controlled by Olympus Slidebook 5.5 software. Colocalization analysis was performed using the colocalization threshold add-on for ImageJ.

#### Cell surface NBCe2 using total internal reflection fluorescence microscopy (TIRFM)

In order to focus on apical membrane-expressed NBCe2 we used TIRFM which allows for selective excitation of the surface-bound fluorophores while having highly suppressed background fluorescence from intracellular and non-bound NBCe2. TIRFM has sub-micron (~70 nm resolution) validating apical presentation of NBCe2. The effect of monensin (10 μmol/L, 24 hr) on NBCe2 apical expression was measured in polarized hRPTCs grown on GEM™ 3D microcarriers, as described previously [49]. Two-color immunofluorescence TIRFM with an imaging depth set at 70 nm from the coverslip surface was used as the imaging depth chosen based on previous work done on α-Na/K/ATPase, that showed a decrease in TIRF when this transporter underwent endocytosis inside the cell and our previous work on NBCe2 in hRPTCs[17].

### Antibodies and their specificities

#### NBCe1 and NBCe2 antibodies

The antibody to NBCe1 (Sigma WH0008671M1) has been well validated in human renal tissue in our previous studies in the RPT [17]. The NBCe2 antibody (Sigma HPA036621) used previously [17] and in the current studies was characterized in the Human Protein Atlas (http://www.proteinatlas.org/ENSG00000188687-SLC4A5/tissue/kidney#imid_20081509). We further verified its specificity using confocal imaging with dual staining for hRPTC-specific marker CD-13 (BD 347837, 1:500 dilution) or HNF4A (goat polyclonal anti-HNF4A, Santa Cruz sc-6556, 1:500). This was performed in cultured primary and immortalized hRPTCs, including overexpressed cell lines or those stably transfected with NBCe2-specific siRNA to knockdown *SLC4A5*, the NBCe2 gene.

#### Western blots

Western blots were performed as previously published [17]. Briefly, detergent-free apical plasma membranes were isolated using sulfo-NHS-SS-biotin as reported previously [59, 60] with the following exceptions: hRPTCs were grown to confluence in normal hRPTC culture media, then EGF was removed, FBS reduced to 0.5%, and rocked at a rate of 1/sec for 3 d to induce polarization. The biotinylated-attached membranes were eluted off the magnetic beads with 100 μL of 70°C 2x sample buffer for 10 min. A sample of cell homogenate was isolated before the biotinylation procedure and represented whole cell homogenate. The whole cell homogenate was prepared by loading equal volumes of sample and 2x sample buffer. Both whole cell and biotinylated samples were separated by SDS-PAGE (10% Tris·HCl polyacrylamide gel) and transferred onto a nitrocellulose membrane by electroblotting. The membrane was blocked in 5% milk in TBS with 0.1% tween-20 (TBS-T) for 2 h at RT and then incubated overnight at 4°C with NBCe2 in Odyssey blocking buffer: NBCe2 (Sigma; 1:500) in 5% milk TBS-T. A second gel was run and transferred, as stated previously. The blot was blocked in Odyssey blocking buffer for 2 h at RT then incubated overnight at 4°C with CD-13 (APN; Santa Cruz; 1:500) and NBCe1 (Sigma; 1:500) in Odyssey blocking buffer. The membrane was subsequently incubated with infrared secondary antibodies (anti-rabbit IR dye 800 and anti-mouse IR dye 680 respectively; both 1:15,000). Immunoreactivity was quantified using the Odyssey Infrared Imaging System. The blot was then stripped using LI-COR nitrocellulose stripping buffer, reprobed with Na^+^,K^+^/ATPase (Epitomics; 1:10,000) primary antibody, and processed as stated previously.

We performed western blot analysis of total whole cell homogenates (WC) and apical membrane fractions from two immortalized hRPTC lines that were grown on Transwell™ membranes. The blot was probed with NBCe2 [17], CD-13 (APN), Na^+^,K^+^/ATPase, PAT-1, and NBCe1 antibodies. CD-13 (APN) was used as an hRPTC microvilli marker and Na^+^,K^+^/ATPase and NBCe1 were used as hRPTC basolateral membrane markers.

### NBCe2 overexpressed and knock-down hRPTCs

**Overexpressed (OE):** A Lentiviral construct (CCSB-Broad Lentiviral Expression Human *SLC4A5* Clone; CloneId: ccsbBroad304_12409) was purchased from Thermo Scientific. The plasmid was packaged into virus with compatible packaging plasmids using HEK-293T cells (Clontech Laboratories). The lentivirus was added to hRTPCs at 30–40% confluence for 18–20 h, then removed and replaced with regular growth medium. After 48 h, the medium was changed to selection medium containing Blasticidin S (InvivoGen; 5 μg/ml).

**Knocked-down (KD):** Validated Mission shRNAi Lentiviral constructs for knocking down the *SLC4A4* gene (Clone ID:NM_003759.1-598s1c1; NM_003759.1-1955s1c1 and NM_003759.1-3183s1c1) or *SLC4A5* gene (Clone ID:NM_021196.3-676s21c1; NM_021196.3-1161s21c1 and NM_021196.2-1989s1c1), as well as a negative shRNA control (Mission Non-Target shRNA control vector; SHC002), were purchased from Sigma-Aldrich and packaged into virus with compatible packaging plasmids using HEK-293T cells (Clontech Laboratories). hRPTCs were transduced, as mentioned above. After 48 h, the selection agent puromycin (Sigma-Aldrich, P7255; 2μg/ml) was added to the media. KD of protein expression was confirmed by western blotting using an anti-NBCe2 antibody (Sigma HPA036621, polyclonal rabbit; 1:500 dilution). The western blots were analyzed using Odyssey software.

#### RT-PCR

Following drug incubation, total RNA was collected using an RNeasy kit (Qiagen). The samples were stored at -80°C prior to RT-PCR. Total RNA was quantified using a NanoDrop Spectrophotometer (Thermo Scientific). The samples were normalized to 100 ng RNA/well on a plastic 96-well plate (Qiagen). The samples were mixed with iScript One-Step RT-PCR kit with SYBR Green (Bio-Rad) using NBCe1, NBCe2, or β-actin Primer Assays (Qiagen).

#### Chromatin immunoprecipitation (ChIP) assay

We performed a ChIP assay to determine whether or not HNF4A bound to or was localized to the region in the human DNA where polymorphisms in *SLC4A5* are located. Approximately 15 million hRPTCs per sample were fixed with formaldehyde (final concentration 1%) and quenched with glycine (final concentration, 125 mmol/L). The hRPTCs were lysed and DNA sheared per manufacturer’s instructions (Thermo Scientific Magnetic ChIP Kit). Protein concentrations were measured by BCA assay (Thermo Scientific). Anti-HNF4A goat polyclonal primary antibody (2 μg, Santa Cruz sc-6556) was used per 100 μg of protein. Protein A/G beads were added to capture the primary antibody and subsequently the samples were washed three times. Following elution, the DNA was purified using Qiagen DNeasy Kit. RT-PCR was performed using primers flanking the SNP site rs7571842 (forward 5’ CCCTTTCCTCTCTCCCTTGT, reverse 5’ ATTTGCAGCAGGTGACTGTG).

#### HNF4A DNA binding assay

100 ng WT or HV *SLC4A5* double-stranded DNA were generated using oligonucleotides matching either the WT G allele or variant A allele, and then annealed and incubated with 10 ng purified V5 myc epitope-tagged HNF4A protein (Origene). ChIP-grade streptavidin magnetic particles were incubated with 100 ng polyclonal mouse anti-myc antibody (Santa Cruz, clone 9E10) for 30 min and washed 3 times with PBS. They were incubated for another 30 min with Alexa647 anti mouse secondary antibody (Invitrogen), washed three times, and the fluorescence measured by microplate fluorometry (PherastarFS). The WT *SLC4A5* oligonucleotides at rs7571842 were 5’ biotinylated-GTCTGTAAAACTAAGGAGGTAATTTGCTGCAACAG and the non-biotinylated complement. The variant *SLC4A5* rs7571842 A allele oligonucleotides were 5’ biotinylated -GTCTGTAAAACTAAGAAGGTAATTTGCTGCAACAG and its non-biotinylated complement. The second WT oligonucleotides at rs10177833 were 5’ biotinylated-ACCCTGGCAATGTGGACACACACCCCATTCAG and the non-biotinylated complement. The 2^nd^ variant (A allele) oligonucleotides at *SLC4A5* rs10177833 were 5’ biotinylated -ACCCTGGCAATGTGGAAACACACCCCATTCAG and the non-biotinylated complement. The positive control consensus HNF4A binding site oligonucleotide was 5’ biotinylated -AGTTCAAAGGTCA.

#### Sodium accumulation assay

The hRPTCs were cultured onto 96-well glass bottom collagen-coated Matrical™ plates (Spokane, WA) at 37°C until they reached 50% confluence. The cells were serum-starved overnight prior to loading with a sodium ion indicator, sodium-binding benzofuran isophthalate (SBFI, 5 μmol/L, Molecular Probes) with 0.04% Pluronic F-127 for 2 h in PBS with calcium and magnesium. The cells were washed twice and allowed to recover at 37°C in serum-free media for 30 min. They were washed two more times with PBS and incubated at room RT with ouabain (100 μmol/L, 10 min) to inhibit Na^+^,K^+^/ATPase activity. The hRPTCs were then placed in a fluorescent plate reader (PherastarFS) and ratiometric readings (340 nm excitation and 510 nm emission/ 380 nm excitation and 510 nm emission) were recorded every 30 sec for 30 min. A 5-min baseline reading was acquired prior to automatic injection of monensin (10 μmol/L). Sodium accumulation was measured as the change in 340/380 ratio at the 30-min time point minus the 5-min average reading prior to monensin injection.

#### Apical to basolateral trans-epithelial hRPTC sodium transport assay

We measured the total sodium transported from the apical to basolateral side of a polarized layer of hRPTCs on a Transwell membrane. The hRPTCs were grown in the polarized state on collagen-coated Transwell inserts in growth media with 5% serum in the lower compartment and 0.5% serum in the upper compartment for 5 days with the media changed daily, and Transwells rocked at a rate of one-half oscillation per sec. After the trans-epithelial electrical resistance (TEER) increased above background at day 5, the plates were switched to serum-free media without growth factors in the upper compartment and 0.5% serum without growth factors in the lower compartment. After the TEER stabilized at 10 days, sodium transport was measured. The solution in the upper compartment was changed to Krebs-Henseleit buffer and the solution in the lower compartment was changed to sodium- and bicarbonate-free media. After 2 h the sodium concentration in the lower compartment was measured by atomic absorption spectroscopy.

#### Sodium bicarbonate-dependent pH recovery assay

Exfoliated hRPTCs obtained from freshly voided urine.

hRPTCs isolated from freshly-voided urine from 3 SS subjects from our clinical study who carried *SLC4A5* variants were compared with 3 SR subjects who carried WT *SLC4A5*. The hRPTCs were cultured overnight (18 h) in serum-free media with 10 μmol/L monensin and loaded as in the sodium accumulation assay described above, with the exception that 2'-7'-bis(carboxyethyl)-5(6)-carboxyfluorescein acetoxymethy ester (BCECF AM, 5 μM, Invitrogen) was the dye used and loaded without Pluronic for 30 min, instead of 2 h. The hRPTCs were then acidified in a CO_2_ incubator without bicarbonate for 30 min. Ouabain was added (100 μmol/L, Na^+^,K^+^/ATPase inhibitor) and the cells were incubated for 10 min. Since the cells showed the same pH recovery response with and without ouabain it was omitted in subsequent experiments. Sodium- and bicarbonate-free media were perfused in the presence of 5-(N-ethyl-N-isopropyl)-amiloride (EIPA, Sigma, 10 μM) to inhibit NHE3 activity[17]. Media with sodium and bicarbonate were then perfused and intracellular pH was measured by time-lapse ratiometric imaging on a spinning disk confocal microscope every 30 sec (485 nm excitation, 530 nm emission/430 nm excitation, 530 nm emission). Ratiometric images were acquired and analyzed using the ratio imaging module of the Slidebook software version 5.5; the initial rates of pH recovery were calculated.

**mmortalized RPTCs obtained from fresh human kidney tissue:** RPTCs isolated from human kidney and identified as *SLC4A5* WT or HV were cultured on thin-bottomed 96-well plates and treated for 24 h with monensin (10 μmol/L) with and without HNF4A antagonists 1-[(2-chloro-5-nitrophenyl)sulfonyl]-2-methyl-1H-benzimidazole, BIM5078, or 2-methyl-1-[(2-methyl-5-nitrophenyl)sulfonyl]-1H-benzimidazole (BI6015, Cayman Chemical, both at 10 μmol/L)[58, 61]. The pH recovery assay was carried out as outlined above, but confocal imaging was not used. Instead, kinetic measurements using the fluorescence plate reader (PherastarFS) were performed. The pH was internally calibrated using the nigericin high potassium calibration method. Two negative controls were used: 1) no sodium in the buffer and 2) when DIDS (Cayman Chemical, 500 μM) [17, 23] was added along with the EIPA.

**Cl^-^/HCO_3_^-^** **exchanger activity**

There is no specific assay for Cl^−^/HCO_3_^−^ exchanger activity. However, pH recovery after removal of CO_2_/HCO_3_^−^ in the absence of sodium is considered a reasonable approximation of Cl^−^/HCO_3_^−^ activity[62]. Cells were loaded with the intracellular pH indicator BCECF AM (5 μM) for 30 min. Sodium-independent HCO_3_^−^ transport was assayed as the initial rate of pHi recovery after an alkaline load (CO_2_/HCO_3_^−^ removal), in the absence of sodium, as described previously [62–65]. The cells were washed free of dye and loaded with CO_2_-equilibrated Krebs–Henseleit solution (25 mmol/L NaHCO_3_) for 30 min. Then, the extracellular solution was replaced with a Krebs-Henseleit NaHCO_3_-free solution and NaCl replaced with choline chloride; pH was measured by a microplate spectrofluorometer and fluorescence monitored every 8 s for ratiometric measurements (485 nm excitation, 530 nm emission/430 nm excitation, 530 nm emission).

**Intrinsic hRPTC intracellular pH buffering** **capacity.**

Intrinsic intracellular buffering capacity was measured by sequentially decreasing concentrations of NH_4_Cl in sodium-, CO_2_-, and bicarbonate-free buffer[63] Sodium-free HEPES buffer contains in mmol/L: HEPES 25, KCl 4.66, MgSO4 1.05, CaCl_2_ 1.35, N-methyl-D-glucamine 148, glucose 11 NH_4_Cl 0, 5, 10, 15, 20, and pH adjusted to 7.4. Intrinsic buffering capacity was measured by labeling cells with the pH sensitive ratiometric dye BCECF as above in a 96-well cell culture plate. The hRPTCs were incubated in sodium-, CO_2_-, and bicarbonate-free HEPES -containing buffer until the intracellular pH stabilized. Then the same buffer containing sequentially decreasing concentrations of NH_4_Cl from 20 mmol/L to 0 at 5 mmol/L intervals were exchanged and read on the microplate reader. At the end of the experiment a single pH measurement was used for internal calibration and compared with complete pH calibration curves performed on each cell line [66].

The intrinsic buffering capacity (β_i_) was calculated from pHi and the concentration of ammonia [64, 65, 67].

These changes were initiated by the removal of the ammonium ions from the extracellular buffer. It has been previously demonstrated that buffering capacity is stable over the range of 6.4–7.2 [17, 65].

### Statistical analysis

Data are expressed as mean ±1SE. Comparisons within and among groups (≥ 3) were made by repeated-measures or factorial ANOVA, respectively, followed by Holm-Sidak or Tukey’s post-hoc tests. Student’s *t*-test was used for 2-group comparisons.

## Results

### Western blots of NBCe1 and NBCe2

**Fig 1:**
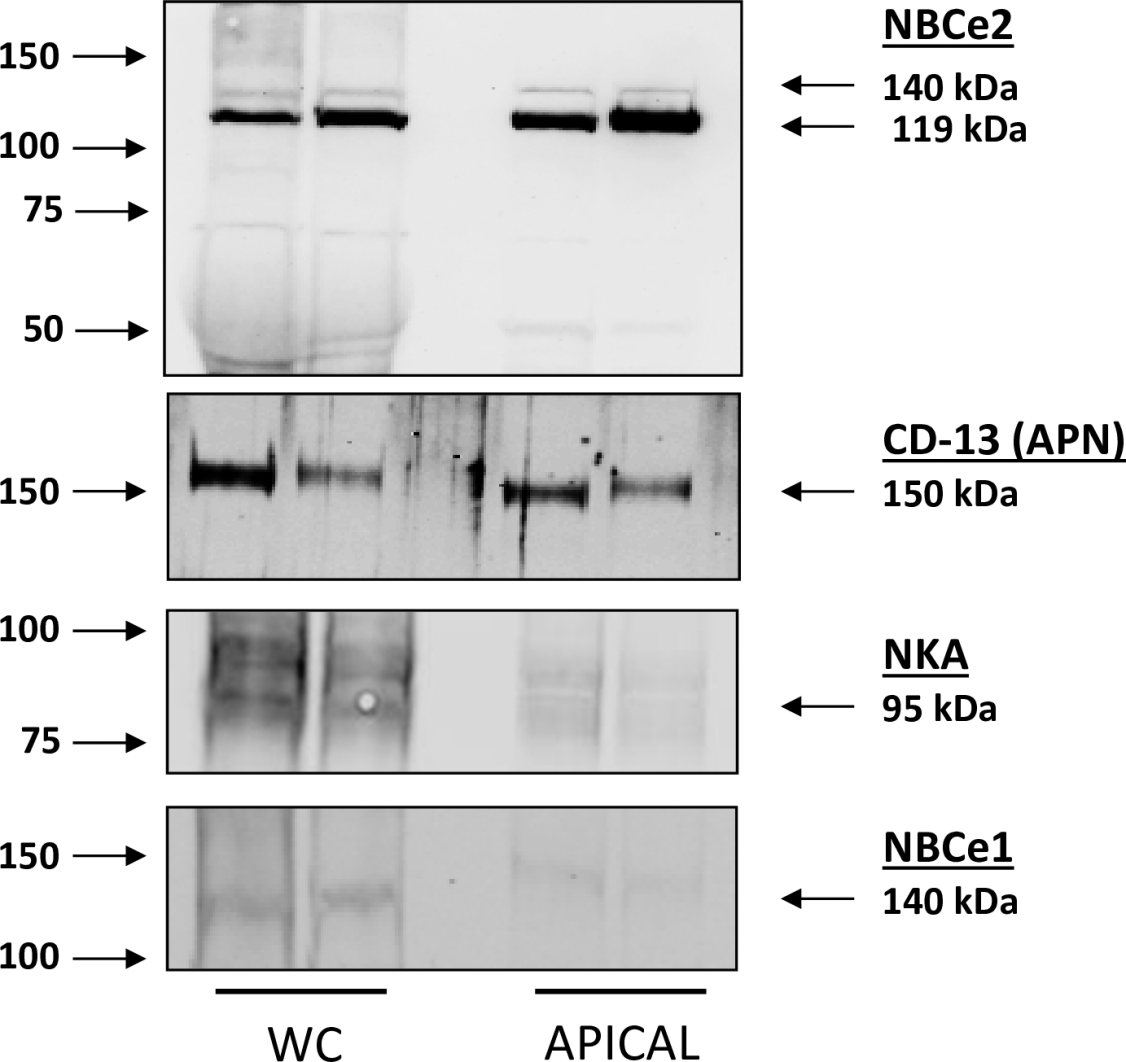
Western blot of whole cell (WC) homogenates and apical membrane fractions from two immortalized hRPTC lines grown in Transwell membranes. Arrows on the left-hand side of the blots indicate the molecular sizes, while the arrows on the right-hand side indicate the molecular sizes of the bands of the proteins of interest. The blot was probed for NBCe2 along with the following membrane markers: CD-13 (APN microvilli marker), Na^+^,K^+^/ATPase (basolateral membrane marker), and NBCe1 (basolateral membrane marker). The results demonstrate that two isoforms of NBCe2 exist in the apical membrane of polarized RPTCs grown on Transwells. The control western blots demonstrate that αNa^+^,K^+^/ATPase (NKA) and NBCe1 only stain weakly in the apical fraction, probably due to some basolateral contamination inherent in this kind of preparation. WC homogenates demonstrate the presence of NBCe2, NBCe1, CD-13, and Na^+^,K^+^/ATPase, as expected.

We performed western blot analysis of total whole cell homogenates (WC) and apical membrane fractions (APICAL) from two immortalized hRPTC lines that were grown on Transwell™ membranes to assure epithelial polarization (**Fig 1**). Arrows on the left-hand side of the blots indicate the molecular marker sizes, while the arrows on the right-hand side indicate the molecular size of the bands of the proteins of interest. The blot was probed for NBCe2 along with the following membrane markers: CD-13 (APN microvilli marker), Na^+^,K^+^/ATPase (NKA),basolateral membrane marker), and NBCe1 (basolateral membrane marker). The results demonstrate that two isoforms of NBCe2 exist in the apical membrane of polarized hRPTCs grown on Transwells™. The control western blots demonstrate that Na^+^,K^+^/ATPase and NBCe1 only stain weakly in the apical fraction, probably due to some basolateral contamination inherent in this kind of preparation. WC homogenates demonstrated the presence of NBCe2, NBCe1, CD-13, and Na^+^,K^+^/ATPase, as expected.

### Effect of Increasing intracellular sodium on the localization of NBCe2 in cultured hRPTCs

**Fig 2:**
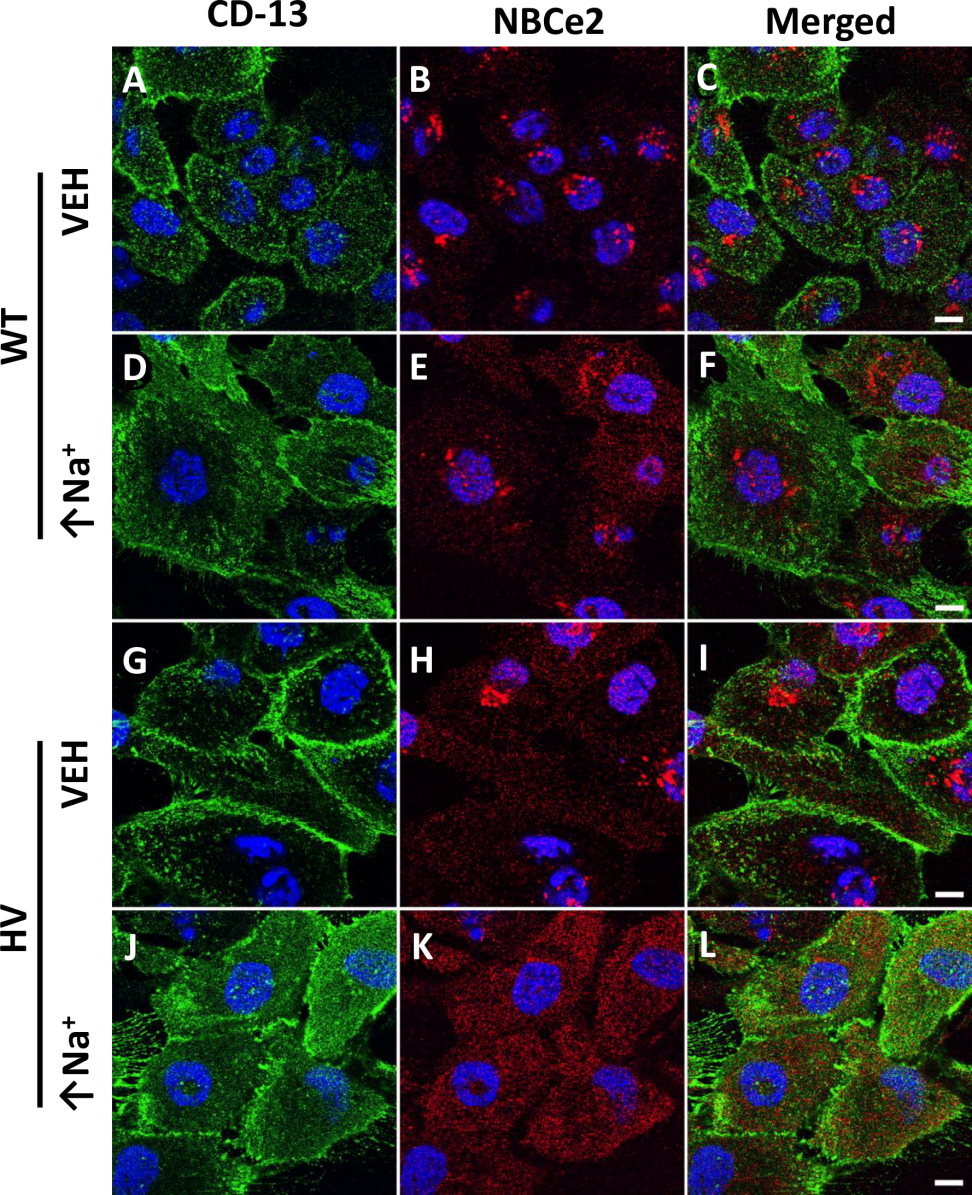
Immunofluorescence localization of NBCe2 in human renal proximal tubule cells (hRPTCs) carrying wild-type (WT) or homozygous variant (HV) *SLC4A5* imaged on Petri dishes using confocal microscopy. CD-13, a specific membrane-bound ectopeptidase present in RPT but not in other nephron segments, in the membrane is stained green using a more photostable fluor (CD-13-Alexa 488 antibody) (**A, D, G,** and **J**), NBCe2 is stained red (**B, E, H**, and **K**), nucleus is stained blue in all the images, including merged images (**C**, **F**, **I**, and **L**). With monensin treatment (10 μmol/L 24 hr) which increases intracellular sodium (↑Na^+^), NBCe2 expression gets more diffuse similar to the apical membrane stain (**E** and **F, K** and **L**), especially for HV cells. Panels C, F, I, and L show merged images of NBCe2 and CD-13; there is increased NBCe2 on the surface in HV cells treated with monensin (**K** and **L**). The scale bar in panel **C**, **F**, **I**, and **L** = 10 μm.

**Fig 3:**
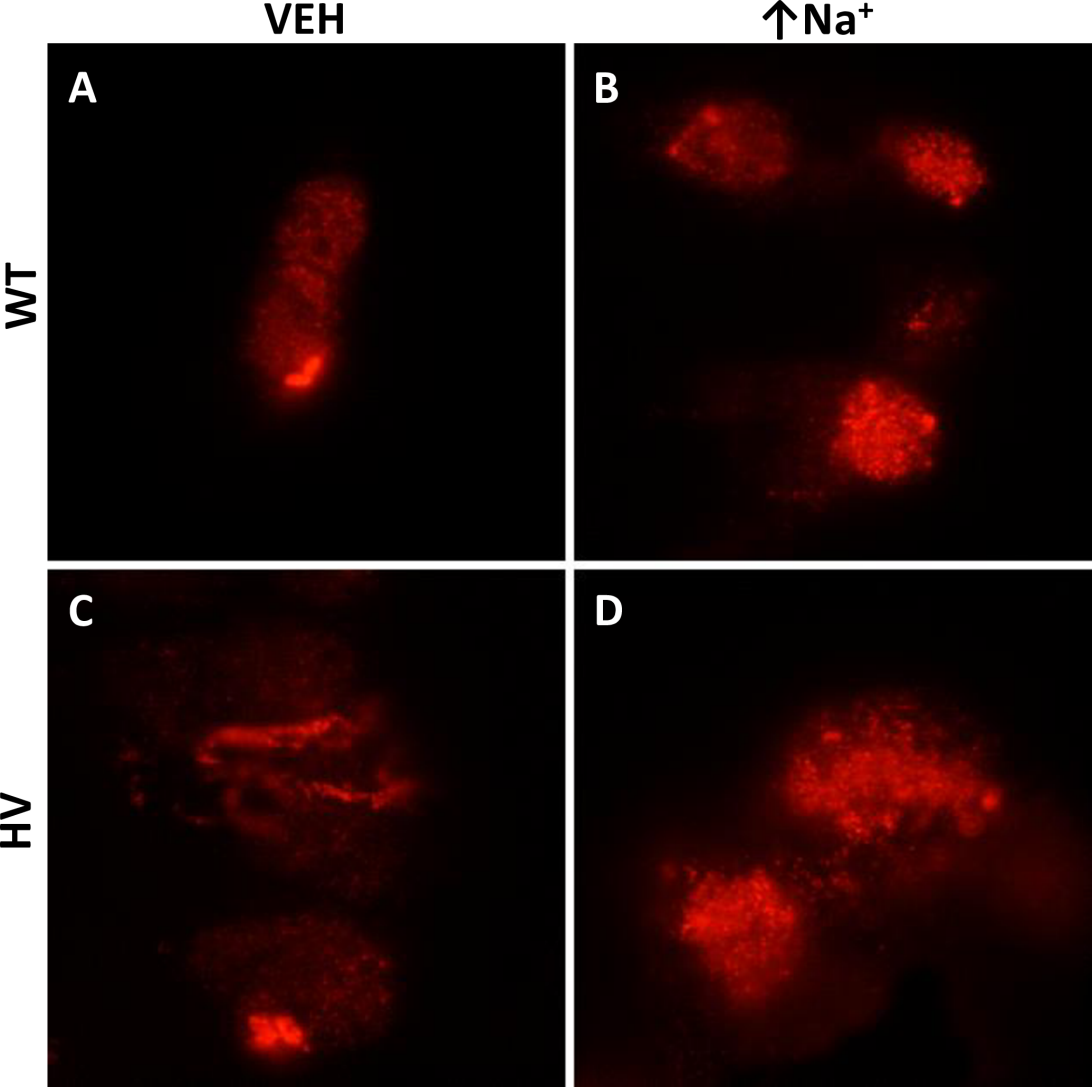
Representative TIRFM images of hRPTCs growing on GEM™ 3D microcarriers. NBCe2 is labeled with a red Alexa 568 fluorescent dye. WT hRPTCs were incubated in vehicle (VEH) (**A**) and imaged at 70 nm into the plasma membrane to determine intramembranous presence of NBCe2. After 24 h of exposure to high intracellular sodium there was no significant increase in membranous localization of NBCe2 as determined by counting punctate fluorescent spots (**B**) (quantified in **Fig 4**). In HV hRPTCs NBCe2 is also expressed in the membrane in VEH treated hRPTCs to a similar level as WT VEH (**C**) but is increased when the HV hRPTCs were exposed to high intracellular sodium (**D**)(quantified in **Fig 4**).

**Fig 4:**
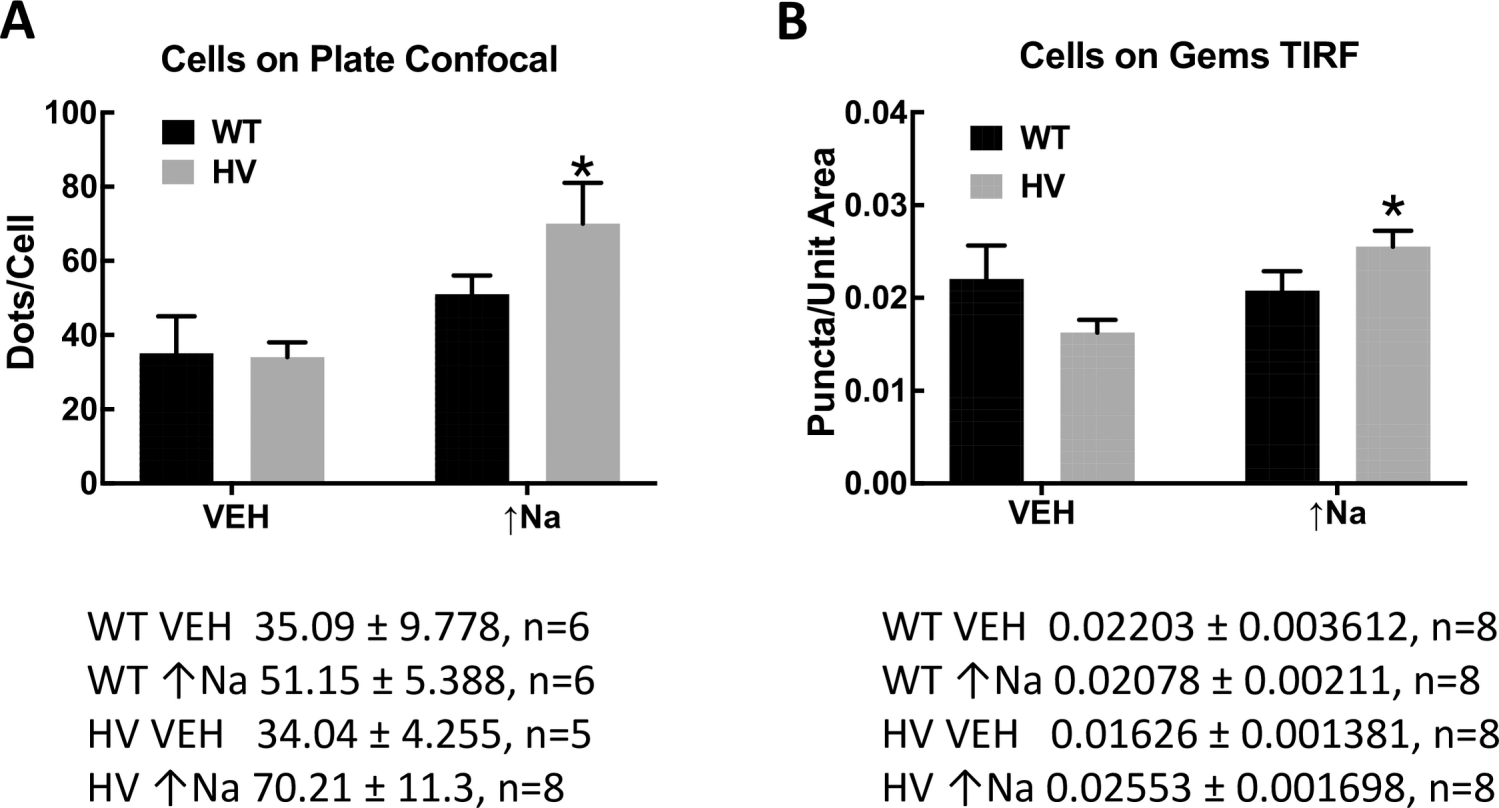
Colocalization analysis of NBCe2 and CD-13 in hRPTC. The number of punctate dots per cell was measured using the Fuji program in both confocal and TIRFM images. We counted punctate dots depicting NBCe2 in hRPTCs grown on Petri dishes (conventional cell culture) using a confocal microscope (**A**) as well as on cells grown as spheroids on novel 3D cell culture hydrogel spheres (GEM™) using our total internal reflectance microscope (TIRFM) (**B)** TIRFM examines only 70 nm at the surface of the apical membrane. Conventional 2D and the novel 3D cell culture demonstrates significantly increased NBCe2 on the apical (CD-13 positive) cell membrane under high salt conditions (↑Na^+^) for *SLC4A5* homozygous variant (HV) cells. (2D Petri dish, P<0.05, two-way ANOVA; 3D GEM™, P<0.05 two-way ANOVA Holm-Sidak test).

Increasing intracellular sodium (monensin, 10 μmol/L) caused an increase in the apical membrane NBCe2 in hRPTCs carrying HV *SLC4A5* but not in those carrying WT *SLC4A5* (**Fig 2**, see merged images **C, F, I, and L**). The apical membrane was marked by CD-13 (APN). We also used TIRFM to image the top 70 nm of the apical cell membrane on cells grown in 3D with their apical membranes being accessible from the media side of the cell (**Fig 3**). Cells grown in 3D are more polarized than cells grown in 2D on Petri dishes. (49) Wild-type control cells did not significantly increase apical membrane expression of NBCe2 when exposed to high sodium conditions (**Fig 4A and 4B)** but HV hRPTCs increased NBCe2 membrane expression from 4.24±0.35% to 11.06±1.72% (P<0.05, N = 3, 2-way ANOVA, Holm-Sidak test) (**Fig 4A and 4B**).

### Effect of *SLC4A5* polymorphisms on basal and intracellular sodium and NBCe2 and NBCe1 expression in hRPTCs

**Fig 5:**
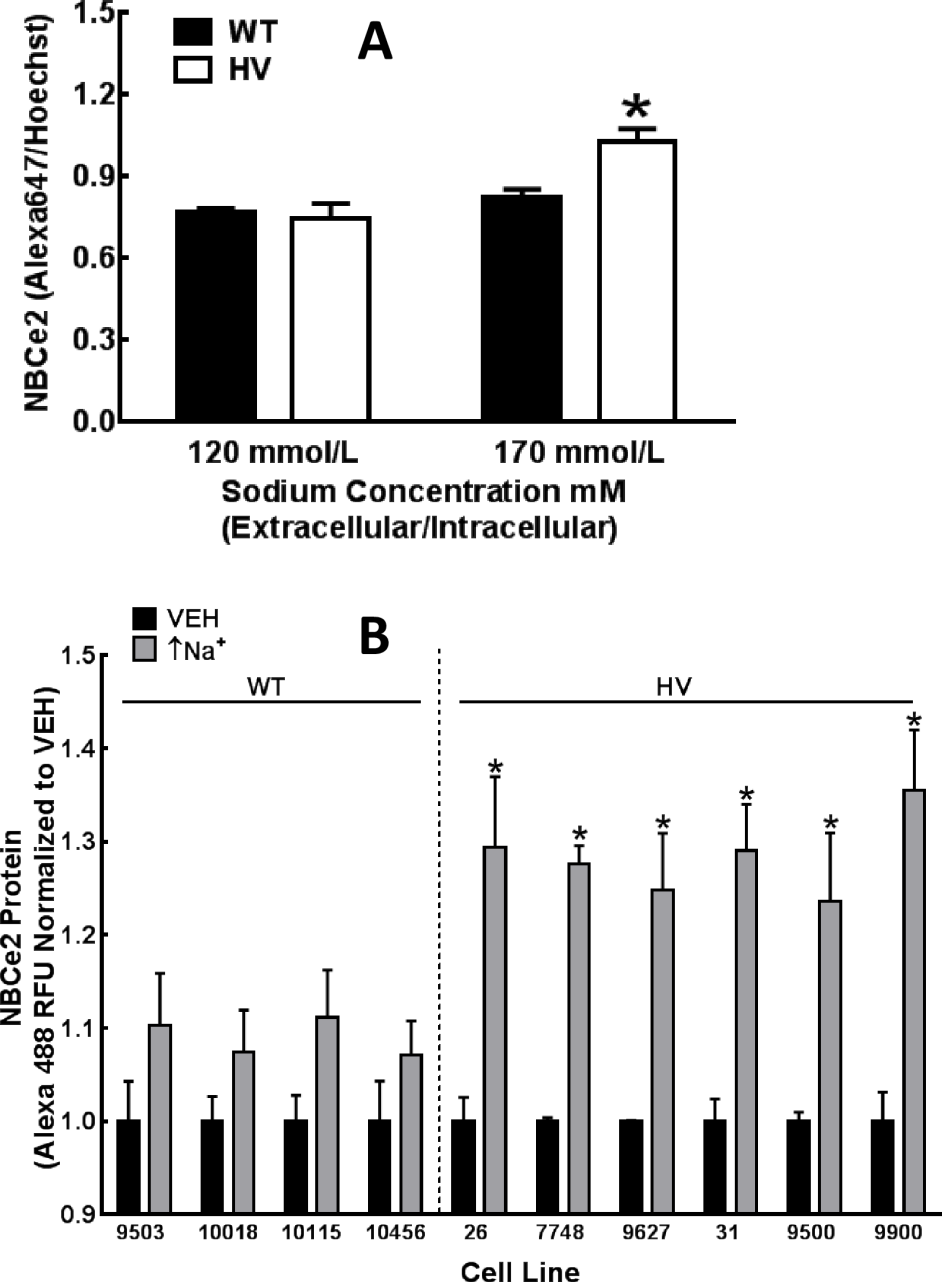
Intracellular sodium-induced NBCe2 protein expression in hRPTCs carrying Wild-Type (WT) or homozygous variant (HV) *SLC4A5*. **A)** Increasing extracellular sodium from 120 to170 mmol/L for 24 h increases intracellular sodium from 6.1±0.3 to 11.3±1.1 mmol/L and increases NBCe2 protein in HV but not WT hRPTCs (N = 4, *P<0.01 vs 120 mmol/L sodium, two-way ANOVA, Holm-Sidak test). **B)** Monensin (10 μmol/L, 24h), which increases intracellular sodium (↑Na^+^) by 6.7±2.3 mmol/L)), slightly increases NBCe2 protein in all four WT cell lines (each cell line from a different individual) but markedly increases NBCe2 expression in all six HV cell lines (each cell line from a different individual) (N = 8–12, *P<0.05 vehicle (VEH) vs monensin (↑Na^+^) with each cell line).

We compared NBCe2 expression in immortalized RPTCs obtained from six individuals who are homozygous for *SLC4A5* SNPs in rs10177833 and rs7571842 (HV) and RPTCs from four individuals who are homozygous wild-type (WT, i.e., no *SLC4A5* SNPs). The basal expression of *SLC4A5* mRNA and NBCe2 protein was similar in hRPTCs with WT and HV *SLC4A5* (**Fig 5**). These observations were similar in immortalized hRPTCs that were or were not serum-starved and hRPTCs in primary culture, indicating that immortalized cells behaved similarly to primary cells. However, increasing extracellular sodium concentration from 120 to 170 mmol/L (which caused an increase in intracellular sodium) (↑Na^+^) [from 6.1±0.3 to 11.3±1.1 mmol/L or a total increase of 5.2±1.1 mmol/L]) increased NBCe2 protein in carriers of HV but not WT *SLC4A5* by 38.00±6.23% (P<0.01, N = 4, 2-way ANOVA, Holm-Sidak test) (N = 4, *P<0.01 vs 120 mmol/L sodium, two-way ANOVA, Holm-Sidak test) (**Fig 5A)**. The increase in intracellular sodium (+6.7±2.3 mmol/L sodium) caused by the non-selective ionophore monensin (10 μmol/L/24 h) slightly increased NBCe2 protein in all four WT *SLC4A5* hRPTCs but markedly increased NBCe2 protein in all six HV *SLC4A5* hRPTCs (**Fig 5B**). The cells that were incubated for 24 h in 170 mmol/L had no increase in apoptosis or necrosis at the 24 h time point (data not shown) but looked phenotypically different from normal cells or cells incubated with the ionophore monensin. Therefore, we favored the use of monensin over increased sodium concentration in the incubation medium in our 24 h experiments.

**Fig 6:**
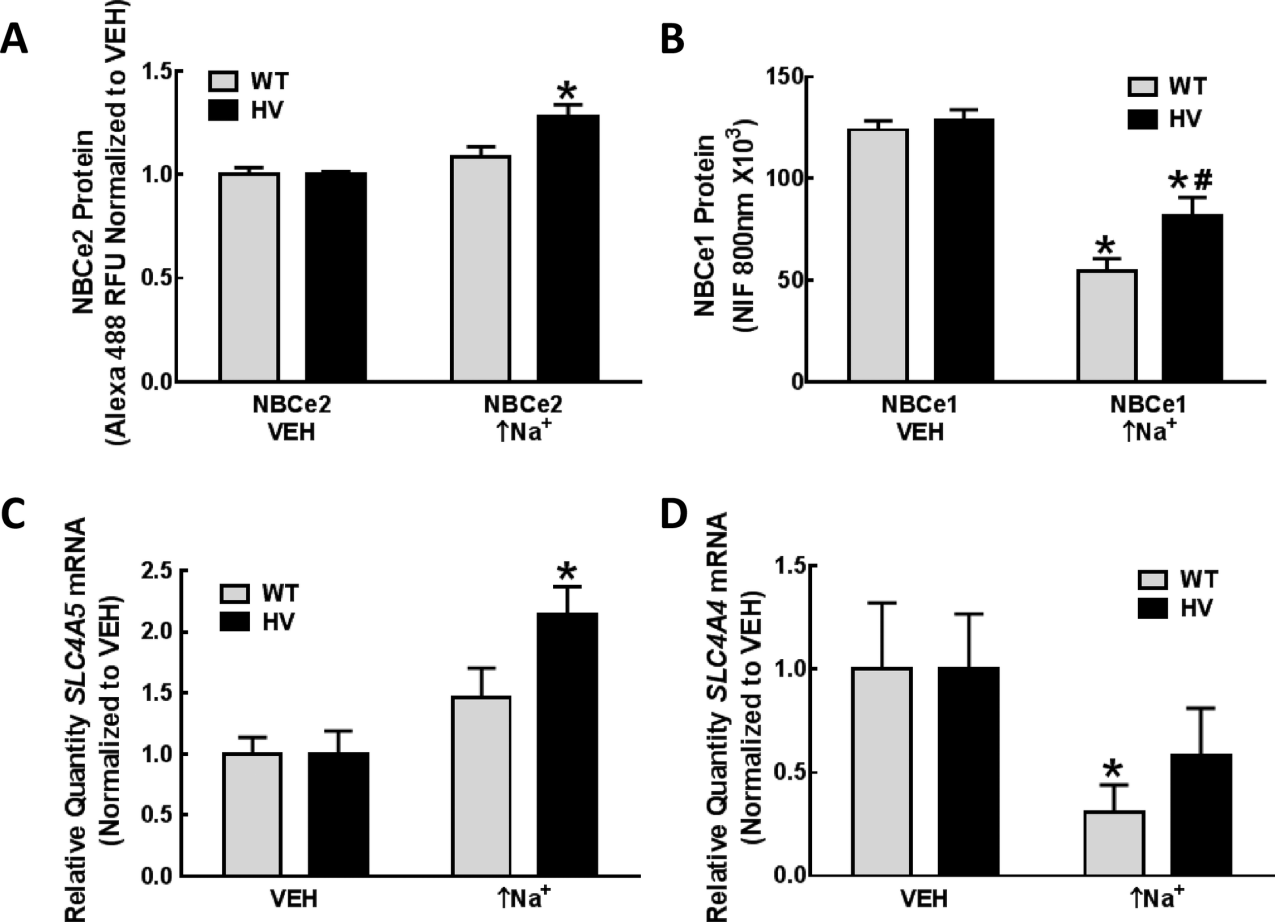
NBCe1 and NBCe2 protein and mRNA expression in hRPTCs carrying wild-type (WT) or homozygous variant (HV) *SLC4A5*. **A) NBCe2 protein.** NBCe2 protein is increased following an increase in intracellular sodium by 6.7±2.3 mmol/L for 24 h (monensin, 10 μmol/L) (↑Na^+^) in HV but not WT *SLC4A5* hRPTCs (N = 12, P<0.0001 vs vehicle (VEH), two-way ANOVA, Holm-Sidak test). **B) NBCe1 protein.** NBCe1 protein is decreased (HV<WT) following an increase in intracellular sodium (monensin, 10 μmol/L, 24 h) (↑Na^+^) in WT *SLC4A5* hRPTCs (N = 36, *P<0.001 vs VEH, two-way ANOVA, Holm-Sidak test) and HV hRPTCs (N = 48, *P<0.05 vs VEH and ^#^P<0.001 vs WT monensin (↑Na^+^), two-way ANOVA, Holm-Sidak test). **C) *SLC4A5* mRNA.** *SLC4A5* mRNA is increased following an increase in intracellular sodium (monensin, 1 μmol/L, 24 h) (↑Na^+^) in HV but not WT *SLC4A5* hRPTCs (N = 9, *P<0.01 vs. others, two-way ANOVA, Holm-Sidak test). **D) *SLC4A4* mRNA.** *SLC4A4* mRNA is decreased following an increase in intracellular sodium (monensin, 1 μmol/L, 24 h) (↑Na^+^) only in WT *SLC4A5* hRPTCs (N = 9, *P<0.05 vs. VEH, one-tailed t-test); the apparent decrease in HV is not statistically significant.

Whereas NBCe2 protein expression was increased by high intracellular sodium (monensin 10 μmol/L) which increases intracellular sodium (↑Na+) by 6.7±2.3 mmol/L), only in HV *SLC4A5* hRPTC cell lines (**Fig 6A**), the same maneuver decreased NBCe1 protein expression in both WT and HV *SLC4A5* hRPTC cell lines (**Fig 6B**). A lower concentration of monensin (1 μmol/L) (↑Na^+^) also increased NBCe2 mRNA only in the HV *SLC4A5* group (**Fig 6C**). The lower concentration of monensin (1 μmol/L) (↑Na^+^) decreased NBCe1 mRNA in the WT group and tended to decrease it in the HV group (**Fig 6D**).

### Effect of dopaminergic and angiotensin stimulation on NBCe2 and NBCe1 expression in hRPTCs

#### NBCe2 expression

D_1_R/D_5_R dopamine receptor agonists (fenoldopam (1 μmol/L) and SKF38393 (1 μmol/L) and angiotensin peptides angiotensin II (Ang II, 10 nmol/L) and Ang III (10 nmol/L), were used to stimulate the hRPTCs for both 3 and 24 h. EC-33 was used to inhibit Ang II peptide metabolism and PC-18 was used to inhibit Ang III peptide metabolism. These compounds did not alter NBCe2 or NBCe1 protein or NBCe2 mRNA expression (data not shown).

**Fig 7:**
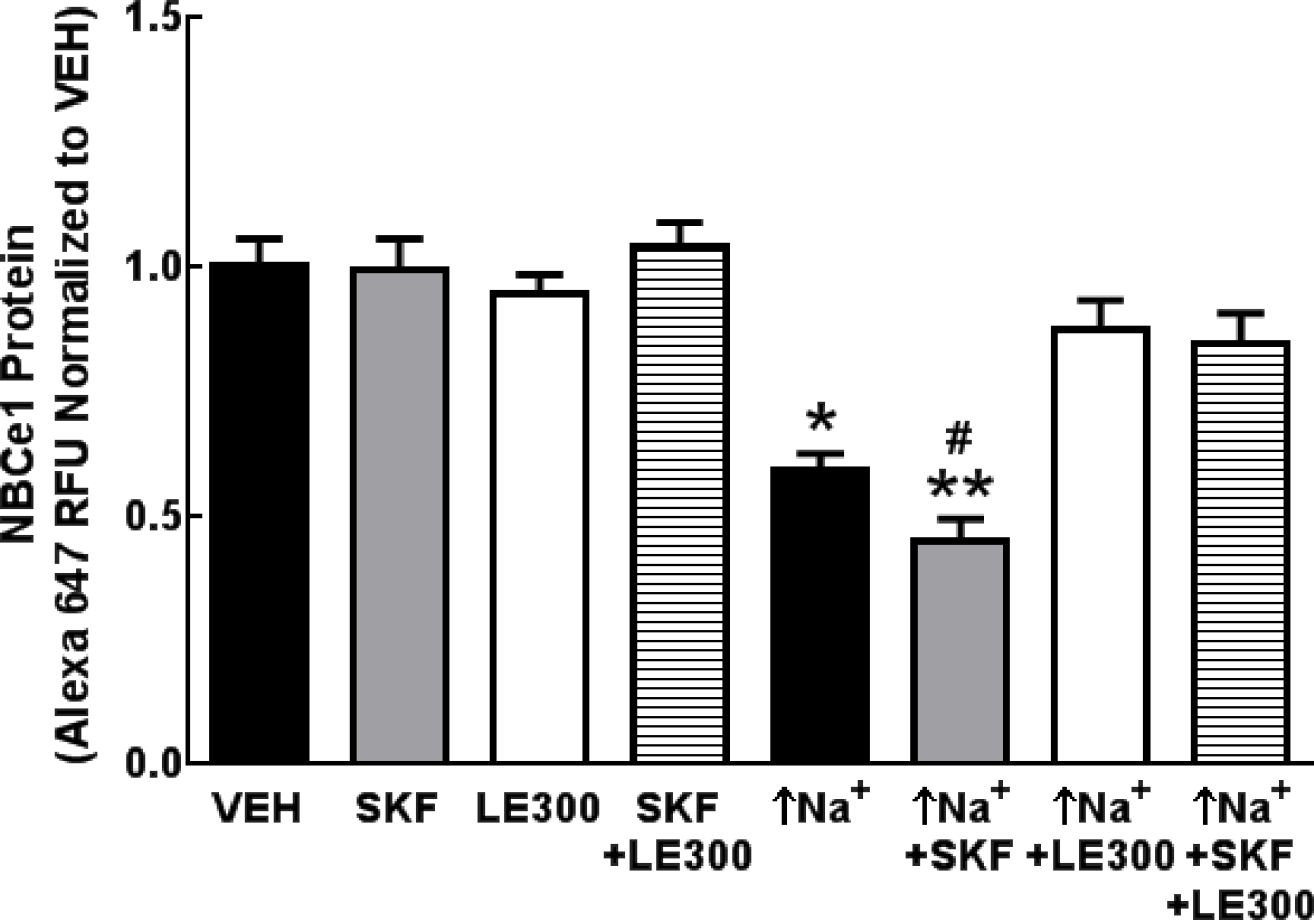
NBCe1 protein expression regulation by D_1_-like (D_1_R and D_5_R) dopamine receptor agonist in hRPTCs carrying wild-type (WT) *SLC4A5*. NBCe1 protein expression is decreased following an increase in intracellular sodium (↑Na^+^) (10 μmol monensin/L, 24 h) (N = 4, *P<0.001 vs VEH, one-way ANOVA, Tukey’s test) in WT *SLC4A5* hRPTC. The combination of monensin (↑Na^+^) and D_1_R/D_5_R agonist SKF38393 (SKF, 10 μmol/L, 24 h) further decreases NBCe1 protein (N = 4, **P<0.05 vs monensin (↑Na^+^), ^#^P<0.001 vs SKF, one-way ANOVA, Tukey’s test) that is blocked by the D_1_-like receptor antagonist LE300 (10 μmol/l, 24 h), which by itself has no effect. NBCe1 protein is not affected by SKF or LE300 in the absence of monensin.

We investigated the effects of dopaminergic stimulation on NBCe1 protein expression in the presence of high intracellular sodium. The D_1_-like receptor agonist SKF38393 (SKF,10 μmol/L) which had no effect on NBCe1 protein under basal conditions enhanced the inhibitory effect of monensin, which increases in intracellular sodium (↑Na^+^) on NBCe1 protein in these WT *SLC4A5* hRPTCs (**Fig 7**). LE300 (10 μmol/L), a D_1_-like (D_1_R/D_5_R) dopamine receptor antagonist, also had no effect on NBCe1 expression under basal conditions, but blocked the decrease in NBCe1 expression induced by monensin (↑Na^+^) and the enhancing effect of the D_1_-like receptor agonist SKF38393 on the inhibitory effect of monensin on NBCe1 protein expression in WT *SLC4A5* hRPTCs. Similar results were obtained in HV *SLC4A5* hRPTCs (not shown).

### Role of HNF4A on the regulation of *SLC4A5* expression in hRPTCs

*SLC4A5* HV SNPs in the Regulome database (RegulomeDB, www.regulomedb.org) indicated that HNF4A binding sites are very close to where the NBCe2 HV SNP sites are located by ChIP sequence data.

**Fig 8:**
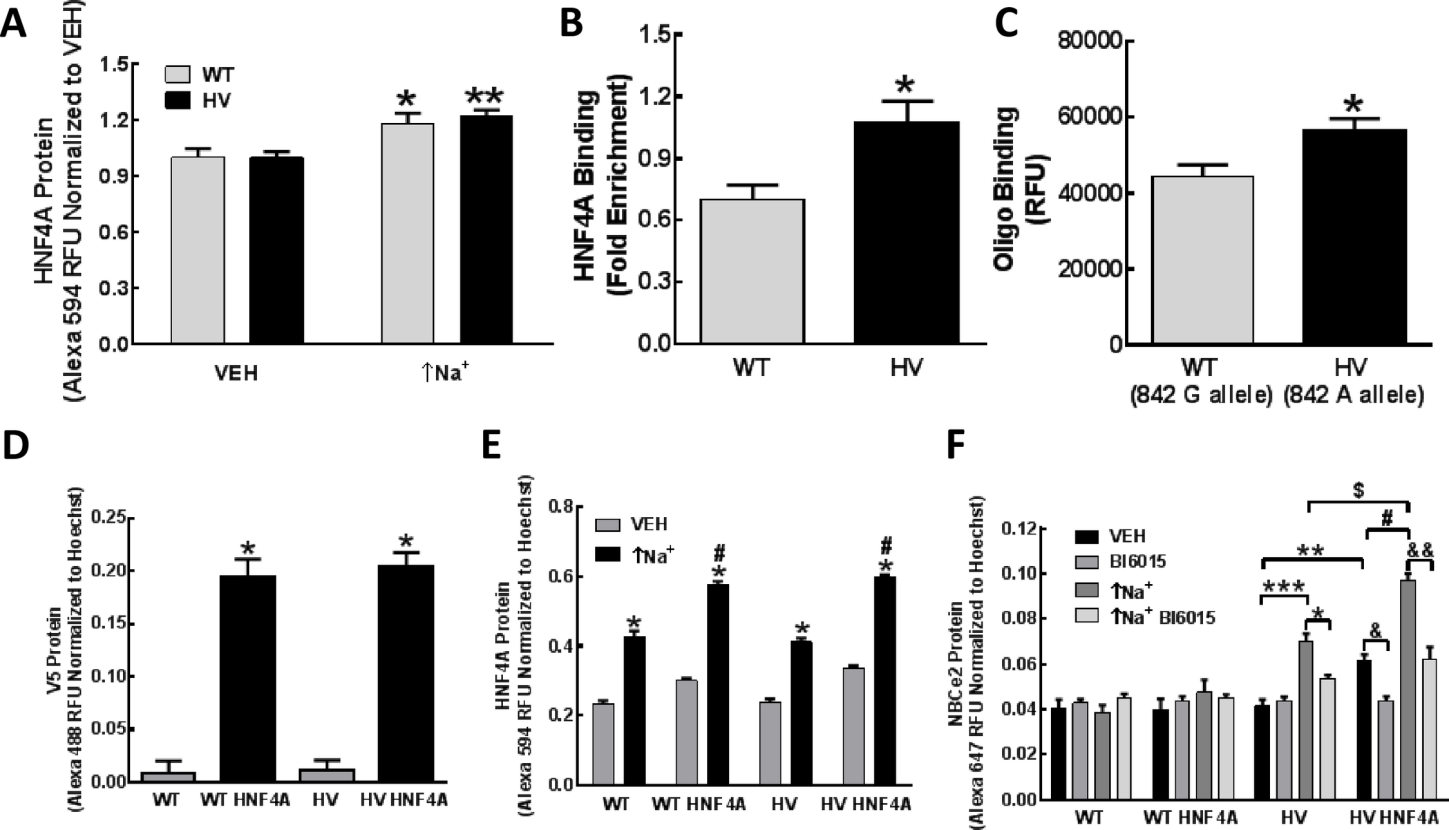
HNF4A expression and binding in hRPTCs carrying wild-type (WT) or homozygous variant (HV) *SLC4A5*. **A) HNF4A protein.** HNF4A protein is increased following treatment with monensin (10 μmol/L, 24 h) which increases intracellular sodium (↑Na^+^) in both WT and HV *SLC4A5* hRPTCs (N = 8, *P<0.05 vs WT VEH; N = 12, **P<0.0001 vs HV VEH, two-way ANOVA, Holm-Sidak test). **B) HNF4A binding at *SLC4A5* SNP site using ChIP.** Approximately 15 million hRPTCs carrying either WT or HV *SLC4A5* were cross-linked and immunoprecipitated with an HNF4A antibody. Magnetic protein A/G was added to capture HNF4A antibody (sc-6556) and any corresponding protein-DNA complex. Samples were then eluted, uncross-linked, and purified for DNA. RT-PCR, using primers flanking the *SLC4A5* SNP site, indicates increased binding of HNF4A to *SLC4A5* in HV hRPTCs. (N = 3, *P<0.05, t-test) **C) *In vitro* oligonucleotide binding assay.** C-myc-tagged HNF4A protein was added to a solution of double-stranded oligonucleotides labeled with biotin. Oligonucleotides consisted of DNA sequences of WT or HV *SLC4A5* alleles. After incubation for 30 min, streptavidin647 was added to label the oligonucleotides; anti-c-Myc antibody was then added followed by magnetic protein A/G to capture HNF4A-oligonucleotide complexes. The samples were washed and read on a microplate reader. HNF4A binding is increased in HV relative to WT sequence (t-test), in agreement with the ChIP data (**Fig 8B**). **D) HNF4A Expression in WT and HV hRPTCs.** V5 Protein in empty vector (control WT, HV) and V5 epitope-tagged HNF4A transfection in 3 WT (WT HNF4A) and 3 HT (HV HNF4A) hRPTC cell lines were measured by in-cell western. V5 protein expression is similar in empty vector-transfected and V5 epitope-tagged HNF4A transfected cells. (N = 9, *P<0.001 vs vector controls, one-way ANOVA, Holm-Sidak test) **E) Total HNF4A expression in empty vector- and HNF4A-transfected WT and HV hRPTCs.** Empty vector control (VEH, WT, HV) and V5 epitope-tagged HNF4A transfected cells (WT HNF4A, HV HNF4A) are equally responsive to monensin (↑Na^+^) treatment. (*P<0.001 vs. WT VEH or HV VEH; ^#^P<0.001 vs others (two-way ANOVA, Holm-Sidak test). **F) NBCe2 expression in WT and HV hRPTCs transfected with empty vector or V5 epitope-tagged HNF4A.** An increase in HNF4A expression leads to increased NBCe2 expression in HV but not WT hRPTCs (**P<0.01HV vs HV HNF4A). The monensin-induced increase in intracellular sodium (↑Na^+^) increases NBCe2 expression in HV (***P<0.001 VEH HV vs monensin (↑Na^+^) HV) and HV HNF4A (^#^P<0.001 VEH HV HNF4A vs monensin (↑Na^+^) HV HNF4A) hRPTCs but to a greater extent in HV HNF4A than VEH HV (^$^P<0.001). The HNF4A blocker, BI6015, prevents the increase in NBCe2 expression in HV HNF4A cells (^&^P<0.001, BI6015 HV HNF4A), and in monensin-treated HV cells (↑Na^+^), without (^*^P<0.05, BI605 HV) or with HNF4A overexpression (^&&^ P<0.05, Bl6015 HV HNF4A). N = 9 in each experiment. All comparisons were made using two-way ANOVA, Holm-Sidak test.

HNF4A protein increased following the increase in intracellular sodium (↑Na^+^) due to monensin (10 μmol/L/24 h) in both WT and HV *SLC4A5* hRPTCs (**Fig 8A**). We, therefore, investigated if HNF4A binds to the rs7571842 *SLC4A5* SNP site using a ChIP assay. We first studied the rs7571842 site because it is the most likely site to bind HNF4A, as shown by our in-silico binding result. We found that HNF4A binding to *SLC4A5* was increased to a greater extent in the HV than WT cell lines (+1.52±0.10-fold, N = 3, P<0.05, t-test, **Fig 8B**). This result was confirmed using an *in vitro* oligonucleotide binding assay that also showed a greater increase in HNF4A binding in HV (+1.27±0.05-fold, N = 3, P<0.05, t-test **Fig 8C**) than WT at the rs7571842 SNP site. Oligo binding of HNF4A to the rs10177833 *SLC4A5* SNP site showed binding above background but did not achieve significance between the WT and HV alleles (data not shown).

In order to investigate the effect of HNF4A over-expression on NBCe2 protein levels, we used V5 epitope-tagged HNF4A lentiviral constructs and established stable cell lines in both WT and HV *SLC4A5* hRPTCs. These cells were then used to test whether or not the transcription factor HNF4A regulates NBCe2 in an *SLC4A5* SNP-dependent manner. The 3 WT and 3 HV independent hRPTC lines from different study participants expressed equal amounts of HNF4A protein (**Fig 8D)**, measured by in-cell western using an antibody to the V5 epitope tag (N = 9, *P<0.001 vs vector WT or HV controls, one way ANOVA, Holm-Sidak test). Total HNF4A protein expression was increased in the transgenic cells compared with vehicle (VEH)-treated vector control cells; monensin (10 μmol/L) (↑Na^+^) increased total HNF4A levels to a similar extent in WT and HV hRPTCs (**Fig 8E**). These data indicate that an increase in intracellular sodium increases HNF4A expression to a similar extent in WT and HV *SLC4A5* hRPTCs.

By contrast, we found that monensin (↑Na+)(10 μmol/L/24 h) increased **NBCe2** expression in vector-transfected HV but not vector-transfected WT *SLC4A5* hRPTCs (**Fig 8F**), indicating that the empty vector construct, per se, does not alter the phenotype of these cells.

In spite of the fact that monensin (10 μmol/L/24 h) increased HNF4A expression to a similar extent in WT and HV cells (**Fig 8A**), HNF4A overexpression increased NBCe2 protein in vehicle-treated HNF4A overexpressing HV *SLC4A5* hRPTCs (HV HNF4A) but not in vehicle-treated HNF4A overexpressing WT *SLC4A5* hRPTCs (**Fig 8F).** Monensin (10 μmol /L/24h) did not increase NBCe2 expression in HNF4A overexpressing WT hRPTCs (WT HNF4A) but increased it in HNF4A overexpressing HV hRPTCs (HV HNF4A) (**Fig 8F**) and to a greater extent than vehicle-treated HNF4A overexpressing HV hRPTCs (HV HNF4A) (**Fig 8F**). These studies suggest that an increase in intracellular sodium allows HNF4A to increase NBCe2 expression in HV but not WT *SLC4A5* cells, presumably related to its increased binding to rs7571842 *SLC4A5* (**Fig 8B and 8C**).

The activity of HNF4A is increased by the presence of rs7571842 *SLC4A5* because the HNF4A inhibitor, Bl6015, prevented the monensin-mediated increase in NBCe2 protein in HV SLC4A5 hRPTCs, regardless of vector transfection (HV) or HNF4A over expression (HV HNF4A) (**Fig 8F**), indicating the importance of an increase in intracellular sodium in the increase in NBCe2 expression in HV *SLC4A5* hRPTCs.

#### Sodium and bicarbonate transport in hRPTCs

In order to determine if the increase in NBCe2 protein in HV *SLC4A5* hRPTCs leads to an increase in sodium transport relative to the WT *SLC4A5* hRPTCs, we measured the transport of several ions in these hRPTCs.

#### Sodium accumulation

**Fig 9:**
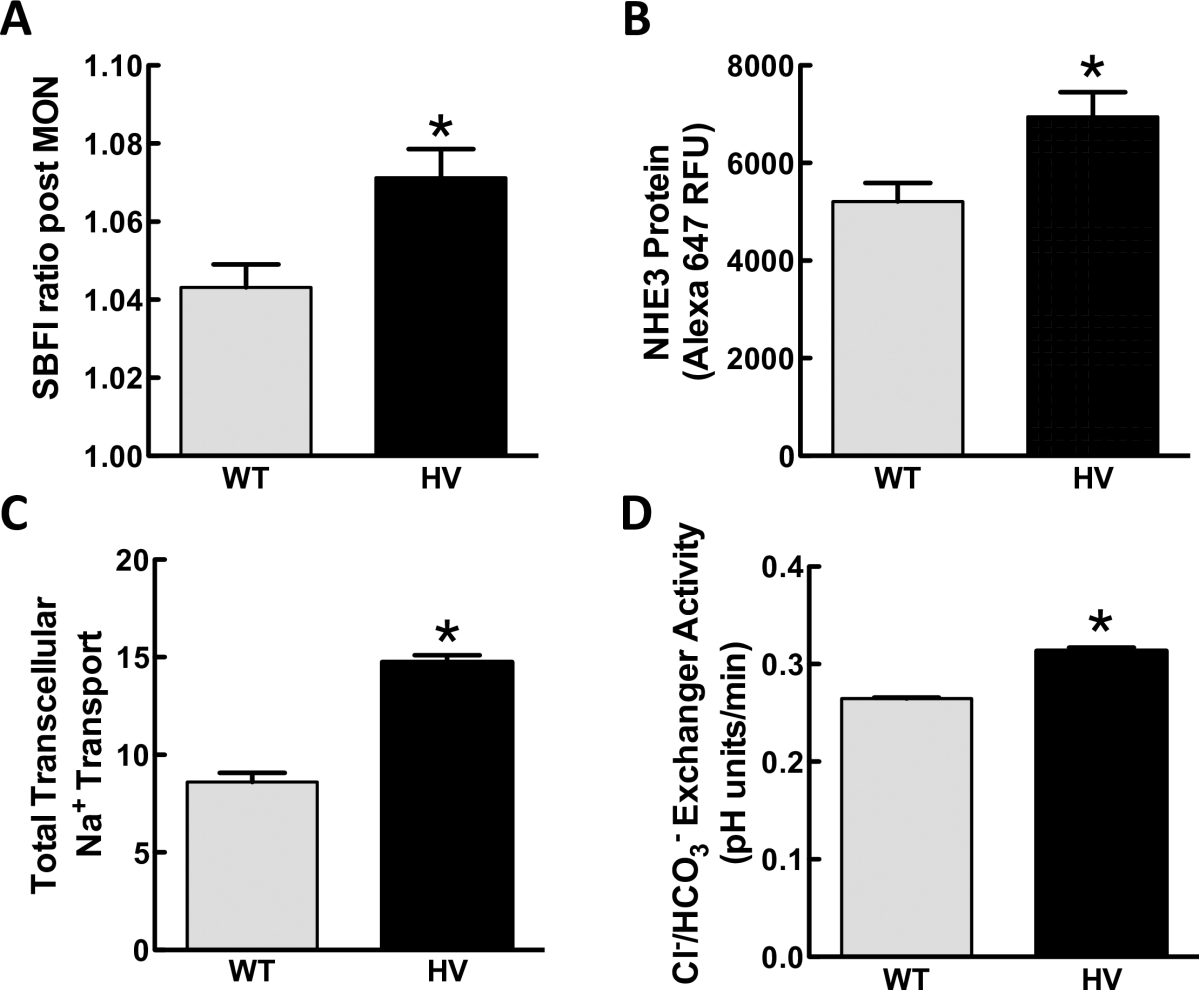
Ion transport assays in cultured hRPTCs carrying wild-type (WT) or homozygous variant (HV) *SLC4A5*. **A) Sodium accumulation assay.** Intracellular sodium was measured in SBFI-loaded hRPTCs in response to monensin (MON, 10 μmol/L, 30 min). Monensin increases F340/F380 SBFI ratio to a greater extent in HV than WT *SLC4A5* hRPTCs (N = 18, *P<0.005, t-test). **B) NHE3 protein expression.** Basal NHE3 protein expression is greater in HV than WT *SLC4A5* hRPTCs (N = 48, *P<0.02, t-test). **C) Transcellular sodium transport in polarized hRPTCs grown in Transwells™.** Six HV *SLC4A5* hRPTC lines derived from 6 different subjects and 4 WT *SLC4A5* hRPTC lines derived from 4 different subjects were grown to confluence on Transwell™ membranes and until a stable trans-epithelial electrical resistance was achieved. Total sodium transport from the upper chamber (luminal) to the lower chamber (basolateral) was measured at 2 h as the amount of sodium measured by atomic absorption of samples taken from the lower chamber. Total sodium transport is greater in HV than WT *SLC4A5* hRPTCs (N = 6–8, *P<0.01, t-test). **D) Cl**^**-**^**/HCO**^**-**^ **exchanger activity.** The rate of pH recovery was measured after an alkaline load (CO_2_/HCO_3_^-^ removal). Six HV *SLC4A5* hRPTC cell lines derived from 6 different subjects (N = 4 experiments per subject) were compared with 4 WT *SLC4A5* hRPTC lines derived from 4 different subjects (N = 4 experiments per subject). HV *SLC4A5* hRPTCs have enhanced Cl^-^/HCO_3_^-^ exchanger activity compared with WT *SLC4A5* hRPTCs; rate of pH recovery is faster in hRPTCs carrying HV than WT *SLC4A5* (*P<0.05, t-test).

We studied the intracellular sodium concentration (measured by SBFI) in cells treated with monensin (10 μmol/L/30 min). The monensin-induced increase in intracellular sodium was higher in three different HV *SLC4A5* hRPTC lines than three different WT *SLC4A5* hRPTC lines (**Fig 9A**). Because basolateral exit of sodium was prevented by inhibiting Na^+^,K^+^/ATPase with ouabain, this result suggested that sodium entry at the luminal membrane is increased that could be due, in part, to increased NBCe2 activity in HV SLC4A5 hRPTCs.

#### NHE3 protein expression

Because the activity of NHE3 and NBCe2 may be tightly linked and protein activity is influenced by protein expression, we measured NHE3 expression in WT and HV *SLC4A5* hRPTC lines. In-cell western assay revealed higher basal levels of NHE3 protein in HV than WT *SLC4A5* hRPTC lines (**Fig 9B**).

#### Total hRPTC sodium transport

Total basal transcellular (luminal to basolateral) sodium transport in polarized hRPTCs grown on Transwell™ membranes was higher in HV than WT *SLC4A5* hRPTCs (**Fig 9C**). Whether or not the increase in total hRPTC sodium transport in HV *SLC4A5* hRPTCs is caused by an increase in luminal sodium and bicarbonate transport could not be determined in this study (**Fig 9A and 9B**) because the basolateral transport due to Na^+^,K^+^/ATPase was not determined.

#### Cl^-^/HCO_3_^-^ exchanger activity

Cl^-^/HCO_3_^-^ exchanger activity was also assessed as the rate of pH recovery after an alkaline load (CO_2_ and HCO_3_^−^ removal).(7, 37) In the basal state, HV *SLC4A5* hRPTCs had increased Cl^-^/HCO_3_^-^ exchanger activity compared with WT *SLC4A5* hRPTCs (**Fig 9D**).

#### Bicarbonate-dependent pH recovery in hRPTCs

**Fig 10:**
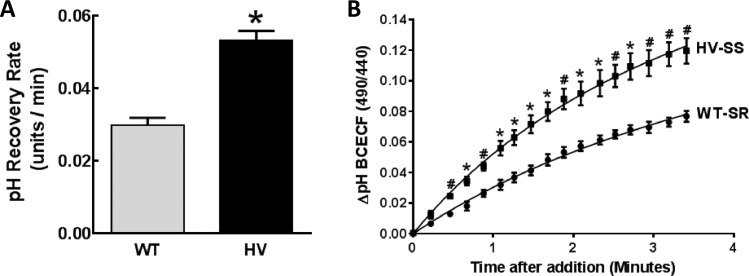
Bicarbonate-dependent pH recovery rate in hRPTCs isolated from urine of salt-sensitive (SS) and homozygous variant (HV) (for the *SLC4A5* gene) or salt-resistant (SR) and wild-type (WT) (for the *SLC4A5* gene) individuals. **A) Bicarbonate-dependent pH recovery rate.** hRPTCs were isolated from the urine of four SS individuals who are HV for *SLC4A5* or from four SR individuals who are WT for *SLC4A5*. The hRPTCs were cultured with monensin (10 μmol/L/24 h) to increase intracellular sodium (↑Na^+^), acidified, and the bicarbonate-dependent pH recovery rate was measured using BCECF in media with EIPA (10 μM) to inhibit NHE3 activity. The ratiometric data obtained by confocal microscopy are greater in hRPTCs from SS (HV) than SR (WT) individuals (N = 4 per group, *P<0.05 vs WT, t-test). **B) Representative recording of bicarbonate pH recovery rate assay.** Exfoliated urinary RPTCs were isolated from two study individuals, one HV-SS and one WT-SR. The sodium bicarbonate recovery rate assay was carried out as in **A** and imaged by ratiometric spinning disk confocal microscopy. The cells were excited at 485 nm, then 440 nm light and the fluorescence emission ratio at 525 nm was recorded every 10 s. Buffers containing sodium at 120 mmol/L and bicarbonate at 25 mmol/L were rapidly perfused at the fourth time point and imaged for 10 min. Because this assay was performed with EIPA to inhibit NHE3 activity, the increase in BCECF is due to increased bicarbonate-dependent pH recovery rate in the HV-SS individual (*P<0.05, ^#^P<0.01 vs SR, N = 3, multiple t-test). Because the monensin-induced increase in sodium (↑Na^+^) downregulated NBCe1 protein to a greater extent in WT than HV *SLC4A5* hRPTCs (**Fig 6B**), but upregulated NBCe2 protein in HV but not WT *SLCC4A5* hRPTCs, the increased bicarbonate-dependent pH recovery in SS with HV *SLC4A5* is most likely due to NBCe2 (**Fig 6A**).

**Fig 11:**
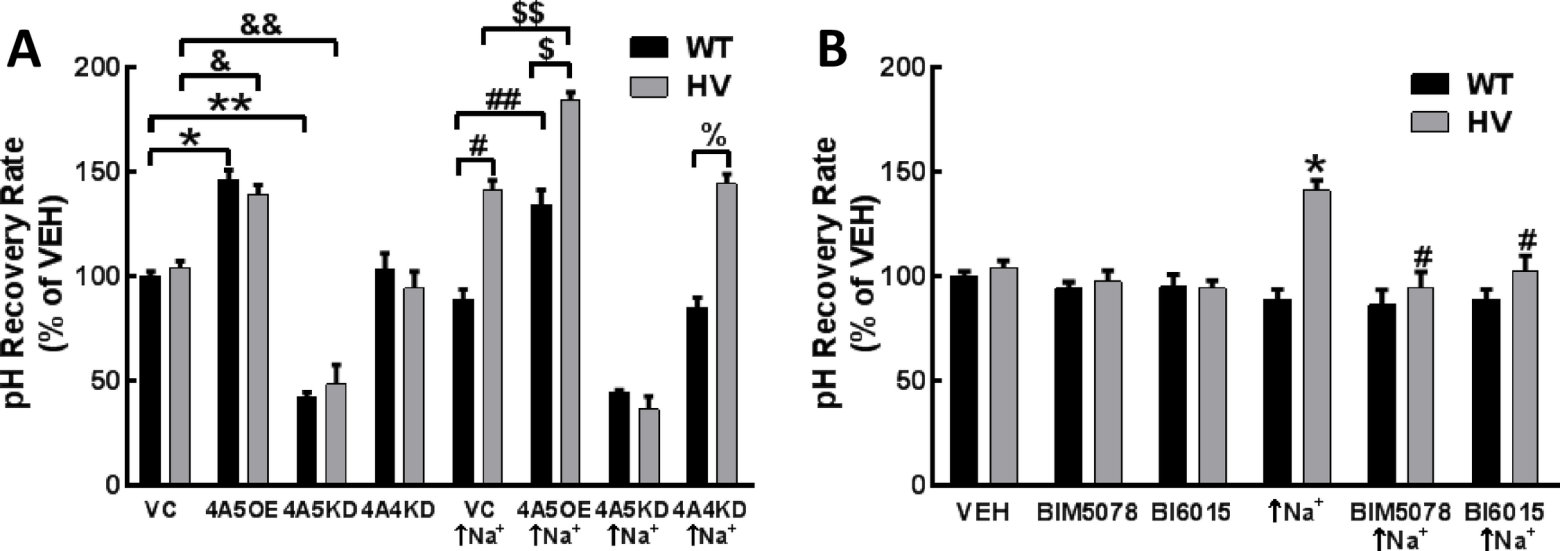
Bicarbonate-dependent pH recovery assays in cultured hRPTCs carrying Wild-Type (WT) or homozygous variant (HV) *SLC4A5*. **A) Bicarbonate-dependent pH recovery**: pH recovery was measured in WT and HV *SLC4A5* hRPTCs expressing empty vector (vector control, VC), overexpressing (OE) SLC4A5 (4A5OE), knock-down (KD) SLC4A5 (4A5KD), and knock-down (KD) SLC4A4 (4A5KD). Bicarbonate-dependent pH recovery is faster in *SLC4A5* OE (4A5OE) (*P<0.001 and ^&^P<0.01) and slower in *SLC4A5* KD (4A5KD) (**P<0.001 and ^&&^P<0.001), relative to VC in both WT and HV *SLC4A5* hRPTCs. By contrast, bicarbonate-dependent pH recovery is not altered in *SLC4A4* KD, relative to VC in either WT or HV SLC4A hRPTCs. Monensin (10 μmol/L, 24 h) treatment that increases intracellular sodium (↑Na^+^) increases pH recovery rate in HV but not WT *SLC4A5* hRPTCs (^#^P<0.001). In *SLC4A5* OE (4A5OE) hRPTCs, pH recovery rate is increased by monensin (10 μmol/L/24 h) (↑Na^+^) in both WT (^##^P<0.001) and HV *SLC4A5* (^$ $^P<0.001) but to a greater extent in the latter than in the former (^$^P<0.001). Knockdown of SLC4A5 (4A5KD) prevents the stimulatory effect of monensin on pH recovery rate in both WT and HV *SLC4A5*. Knockdown of *SLC4A4* (4A4KD) does not affect the increased pH recovery in monensin-treated (↑Na^+^) HV *SLC4A5* hRPTCs (%P<0.05 HV 4A4KD vs WT 4A4KD). **B) Increased bicarbonate-dependent pH recovery rate in monensin-treated HV *SLC4A5* hRPTCs is blocked by HNF4A inhibitors.** Bicarbonate-dependent pH recovery was measured in vehicle (VEH) or monensin (10 μmol/L, 24 h)-treated (↑Na^+^) HV *SLC4A5* hRPTC in the presence of HNF4A inhibitors BIM5078 or BI6015. These inhibitors have no effect when added alone, but either inhibitor completely blocks (n = 4 ^#^P<0.05 vs monensin HV, two-way ANOVA, Holm-Sidak test) the monensin-stimulated (↑Na^+^) (n = 12 *P<0.05, two way ANOVA, Holm-Sidak test) increase in bicarbonate-dependent pH recovery rate in HV hRPTCs.

Sodium bicarbonate transport was measured by the sodium bicarbonate-dependent pH recovery assay(23) in two models of hRPTCs; freshly isolated hRPTCs from human urine (**Fig 10**), and immortalized hRPTCs **(Fig 11).**

#### Exfoliated RPTCS cultured from human urine

To determine if RPTCs from SS human subjects have different bicarbonate-dependent pH recovery compared with RPTCs from SR subjects, we studied cultured hRPTCs (CD-15 positive cells, CD-15 is another marker for hRPTC which was available labeled with magnetic particles allowing hRPTC purification) exfoliated into urine of SS subjects who carry HV *SLC4A5* and SR subjects who carry WT *SLC4A5*. HV *SLC4A5* RPTCs from the urine of SS subjects treated with monensin (10 μmol/L/24 h) had faster bicarbonate-dependent pH recovery than their SR counterparts (**Fig 10A**). Representative recordings from one SS and one SR subject are shown in **Fig 10B.** Because this assay was performed with EIPA to inhibit NHE3 activity, the faster bicarbonate-dependent pH recovery was due to increased bicarbonate transport in SS subjects.

#### RPTCs cultured from human kidneys

Studies were performed in WT or HV *SLC4A5* hRPTCs expressing vector control (VC), overexpressing (OE) *SLC4A5* (4A5OE), knock-down (KD) *SLC4A5* (4A5KD), and knock-down (KD) *SLC4A4* (4A4KD). Relative to vehicle treatment (0.002% EtOH), bicarbonate-dependent pH recovery was faster in *SLC4A5* OE (4A5OE) and slower in *SLC4A5* KD (4A5KD), relative to VC in both WT and HV *SLC4A5* **(Fig 11A)**. By contrast, bicarbonate-dependent pH recovery was not altered in *SLC4A4* KD (4A4KD), relative to VC in either WT or HV *SLC4A* hRPTCs, indicating that either NBCe1 expression does not appreciably affect pH recovery rate in this assay, or that the effect of the KD is masked by compensatory effect of another transporter (**Fig 11A**).

Monensin (10 μmol/L/24 h) (↑Na^+^) treatment of VC cells increased the rate of pH recovery in HV but not WT *SLC4A5* hRPTCs (**Fig 11A**). In *SLC4A5* OE (4A5OE) hRPTCs, pH recovery was increased by monensin (10 μmol/L/24 h) in both WT and HV *SLC4A5* hRPTCs but to a greater extent in the latter than in former hRPTCs (**Fig 11A**). By contrast, knock-down of *SLC4A5* (4A5KD) in monensin-treated hRPTCs slowed pH recovery to the same extent in WT and HT *SLC4A5* RPTCs and similar to that observed in hRPTCs not treated with monensin. These data suggest that an increase in intracellular sodium increases bicarbonate transport only in HV *SLC4A5* hRPTCs. When *SLC4A4* was silenced (*SLC4A4* KD [4A4KD]), monensin (10 μmol/L/24 h) did not affect pH recovery in *SLC4A4* WT cells, similar to the absence of an effect of *SLC4A4* KD (4A4KD) in the vehicle-treated (VC) cells. The increased rate of pH recovery with monensin treatment in HV *SLC4A5* hRPTCs was also not affected by *SLC4A4* KD (4A4KD (**Fig 11A**), indicating that *SLC4A5*, not *SLC4A4*, is responsible for the increase in bicarbonate transport in hRPTCs carrying *SLC4A5* variants.

#### Monensin’s stimulatory effect of bicarbonate-dependent pH recovery in hRPTCs is blocked by HNF4A inhibitors

Two different HNF4A inhibitors (BIM5078 and BI6015) blocked the ability of monensin (↑Na^+^), to hasten the pH recovery in HV *SLC4A5* hRPTCs (**Fig 11B**); these HNF4A inhibitors had no effect when added alone in either WT or HV *SLC4A5* hRPTC (**Fig 11B**). Bicarbonate-dependent pH recovery was completely blocked when the hRPTC were incubated in the absence of sodium or in the presence of DIDS to block anion exchangers, including NBCe1 and NBCe2, indicating the importance of sodium in stimulating bicarbonate transport, presumably due to NBCe2.

#### Effect of HNF4A inhibitors on basal expression of NHE3, PAT1 and Na^+^/,K^+^/ATPase in hRPTCs

**Fig 12:**
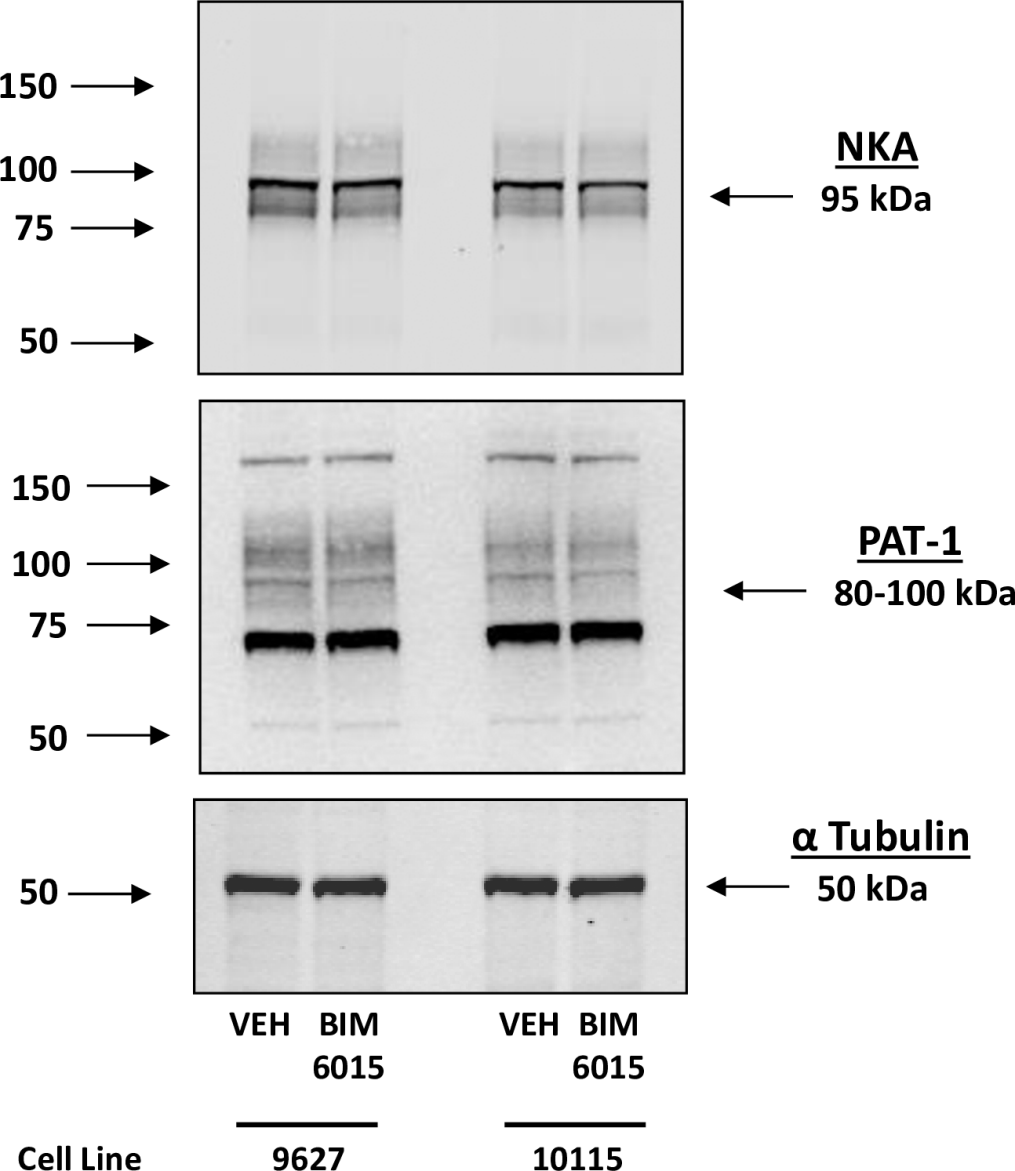
The effect of BI6015 on the expression of Na^+^,K^+^/ATPase (NKA) and the putative anion transporter type 1 (PAT-1). Compared to vehicle control (VEH), neither NKA nor PAT-1 is affected by the addition of the HNF4A inhibitor BI6015. α tubulin was used as a protein loading control.

**Fig 13:**
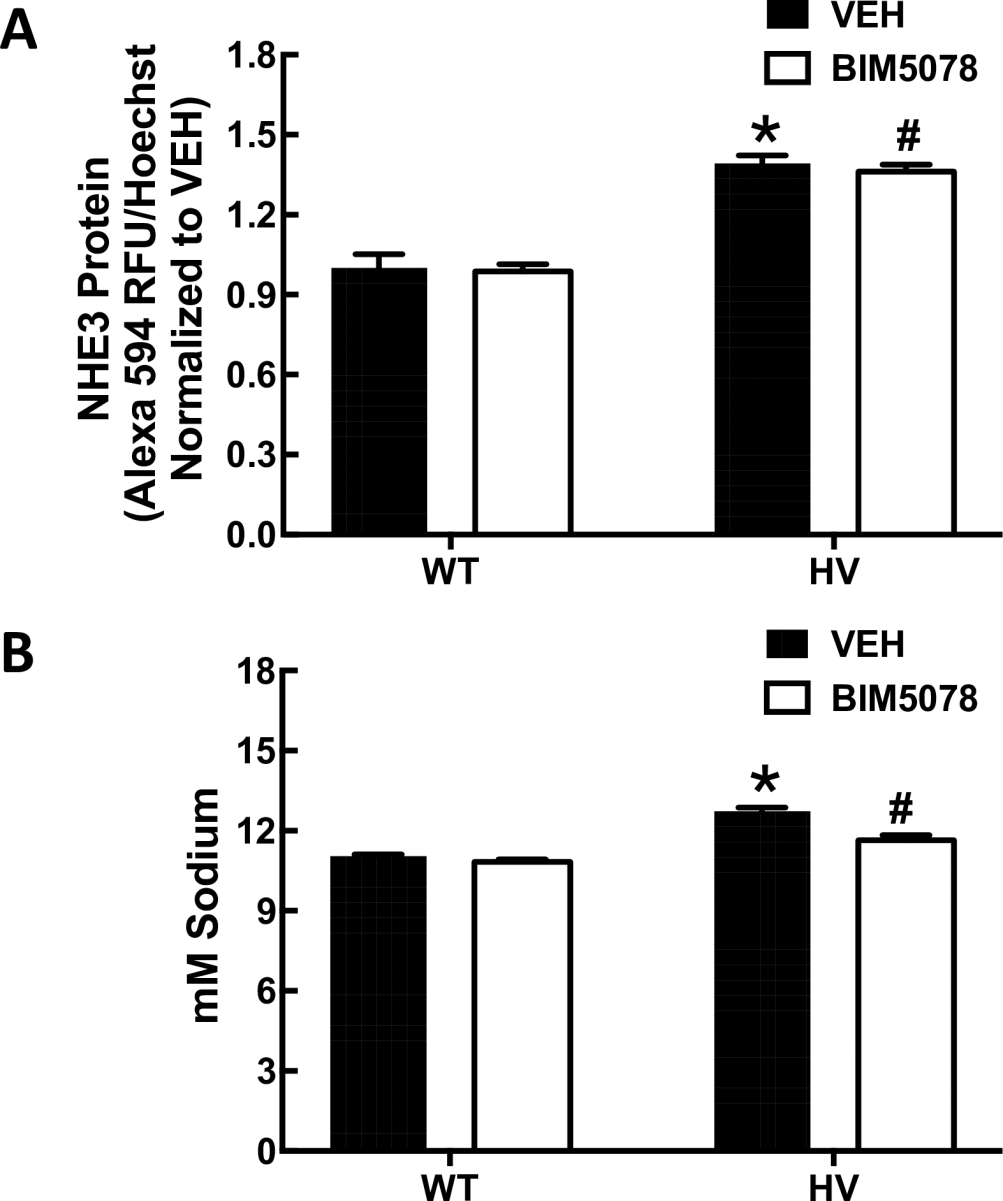
Effect of HNF4A inhibitors on basal expression of NHE3, PAT1 and Na^+^,K^+^/ATPase in hRPTCs. **A) NHE3 protein expression** Basal NHE3 protein expression is greater in HV than WT *SLC4A5* hRPTCs (N = 3, *P<0.01, t-test). Pharmacological inhibition of HNF4A with BIM5078 does not significantly inhibit basal NHE3 protein expression. **B) Sodium influx assay.** Intracellular sodium was measured in SBFI-loaded hRPTCs in response to ouabain (100 μmol/L, 30 min). Ouabain increases F340/F380 SBFI ratio, converted to mM sodium to a greater extent in HV than WT *SLC4A5* hRPTCs (N = 3, *P<0.01, t-test). HNF4A inhibitor (BIM5078, 10 μM, 24 h) inhibits the increased sodium influx in HV hRPTCs (N = 3, #P<0.01 vs HV VEH, two-way ANOVA, Holm-Sidak test) but not in WT hRPTCs.

Because there were no differences between HV and WT hRPTCs in basal HNF4A binding by ChIP assay as well as basal expression of NHE3 protein, sodium transport and Cl-/HCO3 exchange activity, we tested the effect of HNF4A inhibitors under basal conditions. αNa^+^, K^+^/ATPase and PAT1 expressions both with and without the HNF4A inhibitor BIM6015 was measured by western blot (**Fig 12**). The basal protein expression of α-**Na**^**+**^**/,K**^**+**^**/ATPase (NKA)** or PAT1 expression was not changed by HNF4A inhibition. There was a difference in basal NHE3 protein expression between WT and HV hRPTCs, so the effect of inhibiting HNF4A activity with BIM5078, was measured by in-cell western (**Fig 13A**). The basal difference in NHE3 expression was not altered by HNF4A inhibition. Sodium transport is not different between WT and HV hRPTCs under basal condition as well. In order to test whether HNF4A alters this difference, a sodium influx assay was used (**Fig 13B**). Cells were loaded with the sodium sensitive ratiometric dye SBFI and sodium influx was initiated by inhibiting α-**Na**^**+**^**/,K**^**+**^**/ATPase** with ouabain (100 μmol/L) and measuring the amount of sodium accumulation inside the cells. A difference in sodium influx between the two cell types was found, with HV hRPTCs having a slightly higher influx rate than WT hRPTCs. The difference between the two cell types was abolished by the HNF4A inhibitor BIM5078 (10 μmol/L 24 h).

#### Intracellular intrinsic buffering capacity

**Fig 14:**
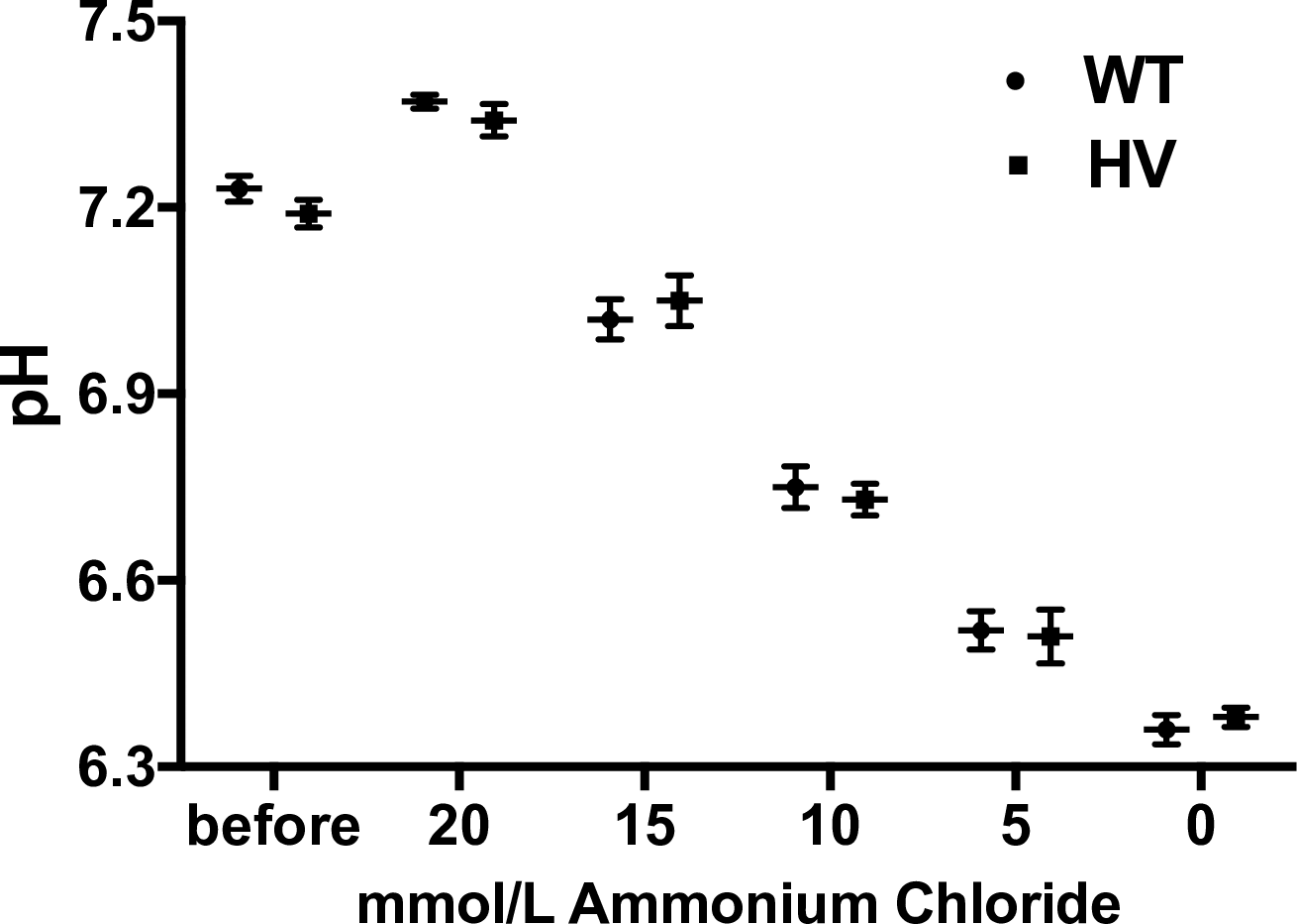
Intracellular pH recovery assay. pH recovery assay in WT and HV hRPTCs shows no significant differences between HV hRPTCs (6 HV hRPTCs each from a different individual) and WT hRPTCs (4 WT hRPTCs each from a different individual), repeated 3 times, a total 18 replicates in HV and 12 replicates in WT hRPTCs **(Fig 14).** These data were transformed mathematically (see Methods) to provide the intrinsic buffering capacity, which demonstrates that there is no significant difference between WT and HV **(Fig 15)**.

**Fig 15:**
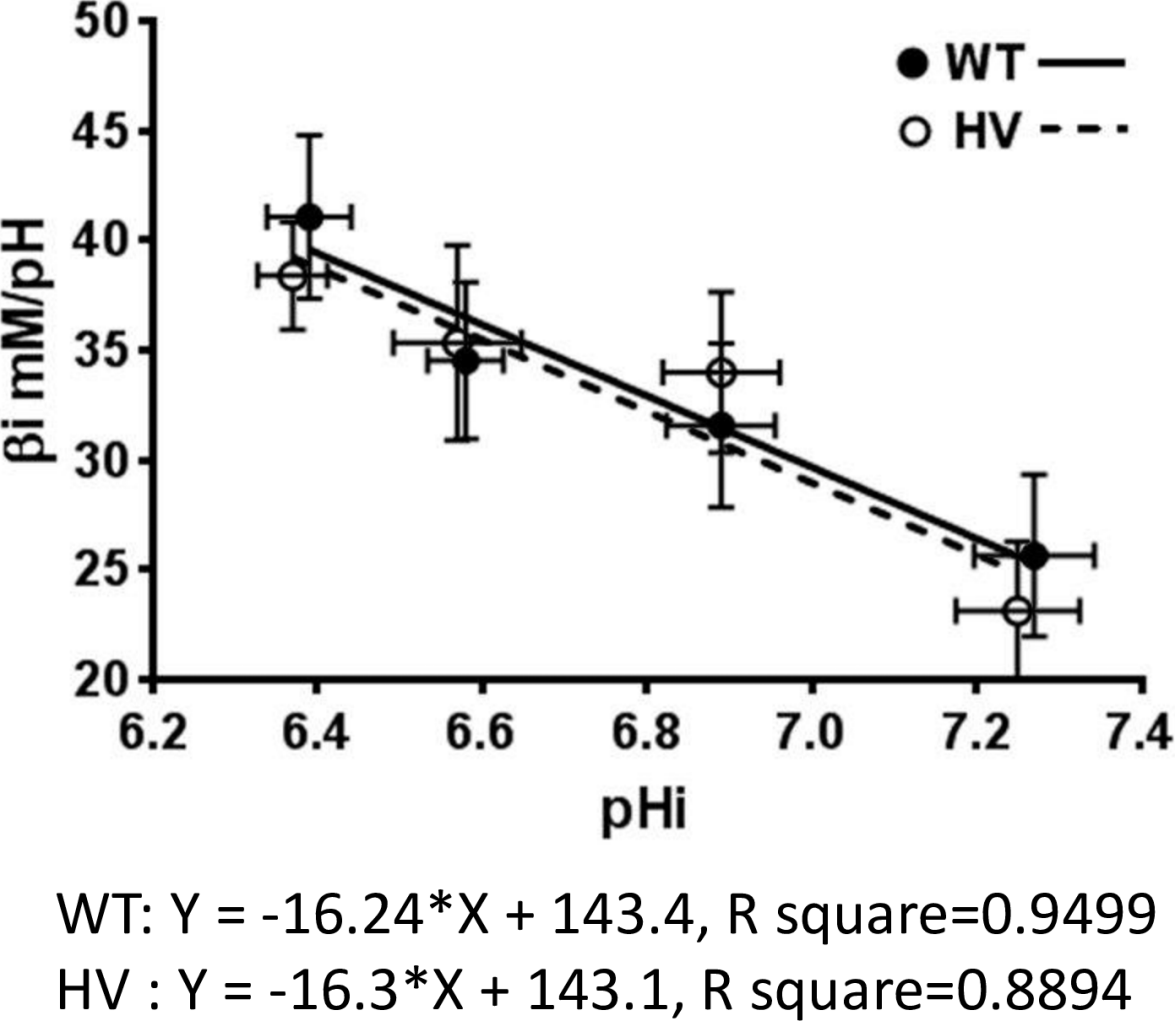
Intracellular intrinsic buffering capacity. Measurement of intrinsic buffering capacity in WT and HV hRPTCs. hRPTCs were labeled with the pH sensitive ratiometric dye BCECF and pH measured by microplate fluorometry by incubating cells in sodium-, CO_2_-, and bicarbonate-free HEPES buffer by sequential incubation in decreasing amounts of ammonium chloride. There are no significant differences in intrinsic buffering capacity between WT and HV hRPTCs (N = 8 per cell type).

We performed pH recovery assays in WT and HV hRPTCs and found no significant difference between HV (6 HV hRPTCs each from a different individual) and WT RPTCs (4 WT hRPTCs each from a different individual), repeated 3 times, a total 18 replicates in HV and 12 replicates in WT hRPTCs **(Fig 14).** These data were transformed mathematically (see Methods) to provide the intrinsic buffering capacity, which showed that there was no difference between WT and HV specimens **(Fig 15)**.

#### Proposed models of ion transport in hRPTC with HV *SLC4A5*

**Fig 16:**
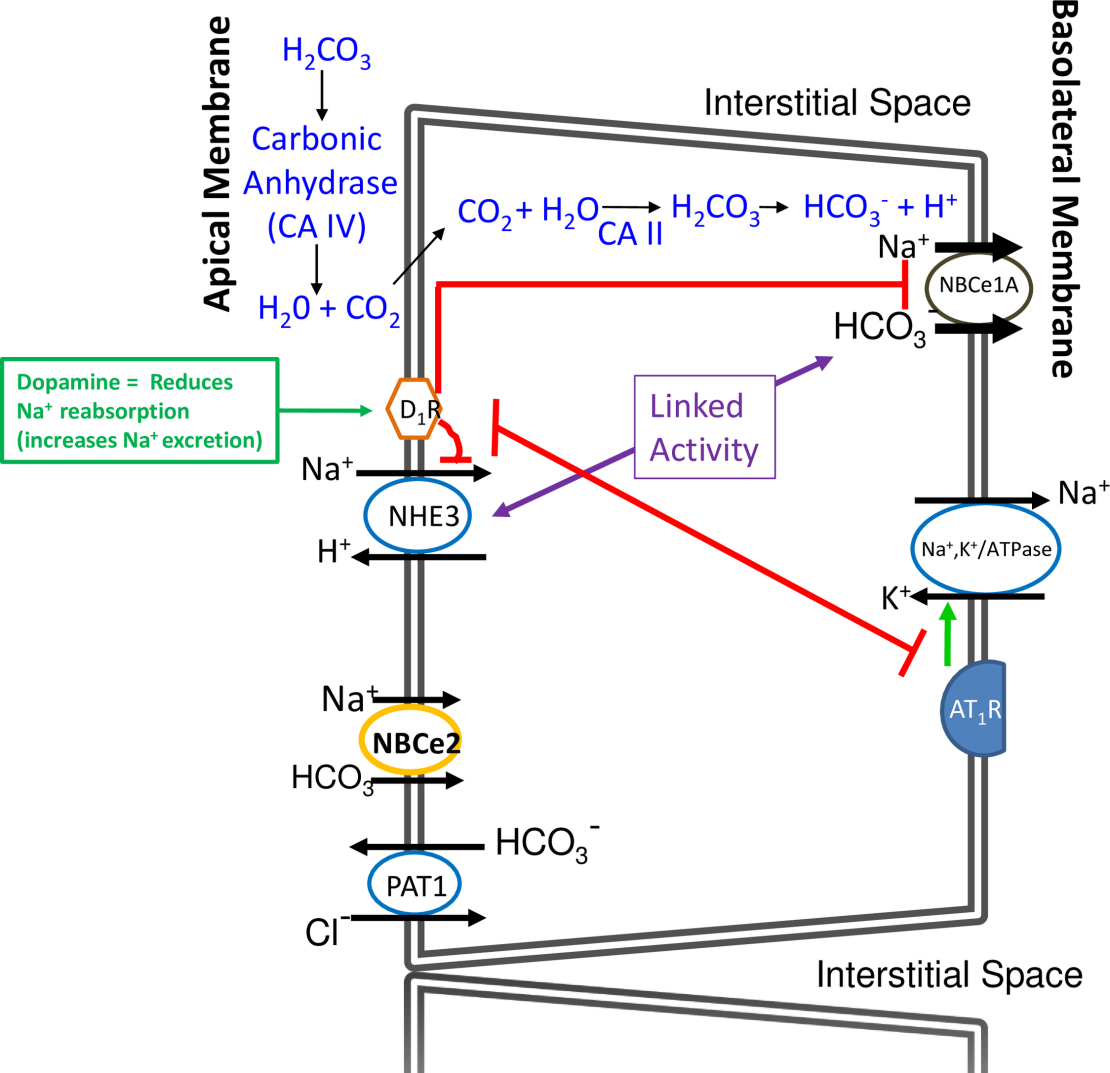
Models of ion transport in hRPTCs. This is a model of the hRPTC with the apical (brush border, facing the lumen) (left hand side) and the basolateral side (right hand side). The principal ion transporters and some of the receptors that regulate them are shown. Starting at 11 o’clock in blue is shown the classic pathway for transporting bicarbonate (HCO_3_^-^) into the cell. Filtered NaHCO_3_ dissociates into Na^+^ and HCO_3_. HCO_3_^-^ in the luminal fluid and H^+^ secreted into the lumen form H_2_CO_3_. Carbonic anhydrase type 4 (CA IV) in the luminal membrane catalyzes the conversion of H_2_CO_3_ to H_2_O and CO_2_.CO_2_ diffuses inside the hRPTC where intracellular carbonic anhydrase type 2 (CA II) catalyzes the conversion of CO_2_ and H_2_O into H_2_CO_3_ which then dissociates into HCO_3_^-^ and H^+^. At 9 o’clock is NHE3 which exchanges one Na^+^ from the lumen with one H^+^ inside the hRPTC. At 7 o’clock HCO_3_^-^ Cl^-^ exchanger (PAT1) is depicted which exchanges luminal Cl^-^ with cytoplasmic HCO_3_^-^. At 3 o’clock is depicted NBCe1 at the basolateral membrane which electrogenically transports 2–3 Na^+^ and one HCO_3_^-^ into the basolateral space. At 4 o’clock is Na^+^, K^+^/ATPase which pumps 3 Na^+^ out of the cell into the blood stream and pumps in 2 K^+^ inside the cell The topic of this manuscript deals with NBCe2, drawn at 8 o’clock. Under a normal sodium load it plays a minor role in Na^+^ and HCO_3_^-^ transport into the hRPTC. There are various plasma membrane receptors that regulate some of these transporters/ exchanger/pumps. The dopamine-1 receptor (D_1_R) (ten o’clock) when stimulated with dopamine (green box) inhibits (red lines) both NHE3 and Na^+^, K^+^/ATPase (without the red line, for simplicity) activities resulting in reduced Na^+^ reabsorption and increased Na^+^ excretion. The AT_1_R (5 o’clock) increases Na^+^, K^+^/ATPase activity (green arrow) resulting in increased Na^+^ reabsorption. An increase in intracellular Na^+^ increases Na^+^, K^+^/ATPase activity (5 o’clock) that is abetted by AT_1_R (green arrow) resulting in increased Na^+^ transport from inside the cell to the basolateral space. The D_1_R and AT_1_R oppose each other. The D_1_R inhibits the AT_1_R, resulting in reduced Na^+^ transport. We showed that NBCe2 is not affected by stimulation of the D_1_R or AT_1_R. Under basal conditions, NBCe1 is more active than NBCe2 (depicted as relatively larger directional transport arrows).

**Fig 17:**
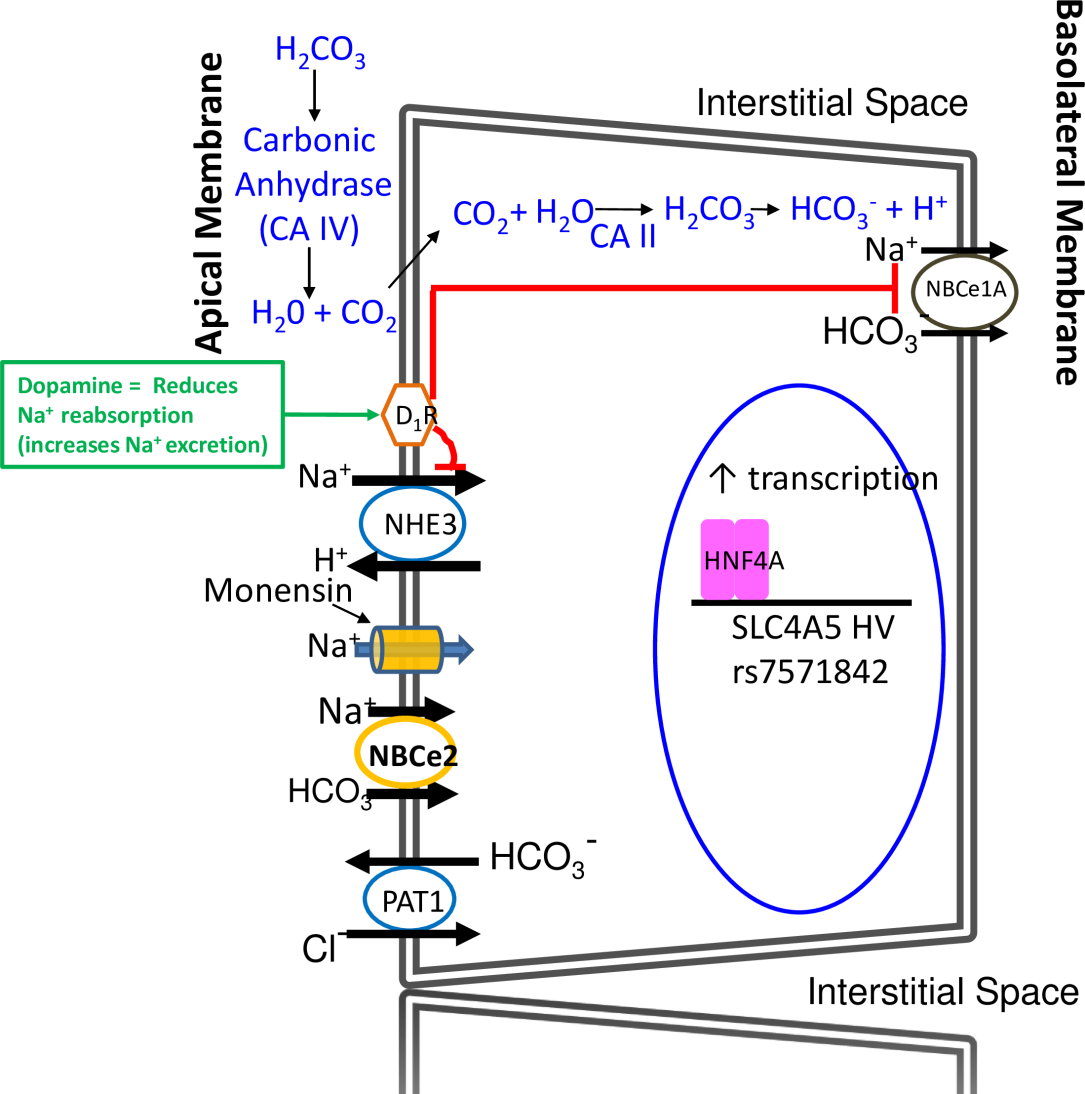
Model of ion transport in hRPTCs with HNF4A binding to *SCL4A5* promoter. In a hRPTC containing the SNP rs7571842 in *SCL4A5* the model changes to what is depicted in **Fig 17.** Increasing intracellular Na^+^ concentration with high extracellular Na^+^ concentration or monensin (9 o’clock) increases NBCe2 mRNA, protein, and activity, while only marginally attenuating the protein and activity of NBCe1 (2 o’clock) in hRPTCs with SNPs in NBCe2. This results in a net increase in Na^+^ transport into the basolateral space. PAT1 (7 o’clock) activity increases because of an increase in intracellular bicarbonate (7 o’clock). NHE3 (9 o’clock) activity also increases because the increase in NBCe2 activity increases intracellular H^+^ following the conversion of transported HCO_3_^-^ to H_2_CO_3_ and its dissociation to H^+^ and HCO_3_^-^ resulting in a further increase in sodium reabsorption. An increase in HNF4A binding to the rs7571842 *SLC4A5* increases its expression.

The principal ion transporters and some of the receptors that regulate them are shown in the models in **Figs 16 and 17**. In **Fig 16, s**tarting at 11 o’clock in blue is shown the classic pathway for transporting bicarbonate (HCO_3_^-^) into the cell. Filtered NaHCO_3_ dissociates into Na^+^ and HCO_3_^-^. HCO_3_^-^ in the luminal fluid and H^+^ secreted into the lumen form H_2_CO_3_. Carbonic anhydrase type IV (CA IV) in the luminal membrane catalyzes the conversion of H_2_CO_3_ to H_2_O and CO_2_; CO_2_ diffuses inside the hRPTC where intracellular carbonic anhydrase type 2 (CA II) catalyzes the conversion of CO_2_ and H_2_O into H_2_CO_3_ which then dissociates into HCO_3_^-^ and H^+^. At 9 o’clock is NHE3 which exchanges one Na^+^ from the lumen with one H^+^ inside the hRPTC. At 7 o’clock is depicted a HCO_3_^-^ Cl^-^ exchanger (PAT1) which exchanges luminal Cl^-^ with cytoplasmic HCO_3_^-^. At 3 o’clock is depicted NBCe1 at the basolateral membrane which electrogenically transports 2 Na^+^ and one HCO_3_^-^ into the basolateral space. At 4 o’clock is Na^+^,K^+^/ATPase which pumps 3 Na^+^ out of the cell into the basolateral space and pumps in 2 K^+^ inside the cell. The topic of this manuscript deals with NBCe2, drawn at 8 o’clock. Under a normal sodium load it plays a minor role in Na^+^ and HCO_3_^-^ transport into the hRPTC.

There are various plasma membrane receptors that regulate some of these transporters/ exchangers/pumps. The dopamine-1 receptor (D_1_R) (ten o’clock) when stimulated with dopamine (green box) inhibits (red lines) both NHE3 and Na^+^,K^+^/ATPase (without the red line, for simplicity) activities resulting in reduced reabsorption and increased Na^+^ excretion. The AT_1_R (5 o’clock) increases Na^+^,K^+^/ATPase activity (green arrow) resulting in increased Na^+^ reabsorption. An increase in intracellular Na^+^ increases Na^+^,K^+^/ATPase activity (5 o’clock) that is abetted by AT_1_R (green arrow) resulting in increased Na^+^ transport from inside the cell to the basolateral space. The D_1_R and AT_1_R oppose each other. The D_1_R inhibits the AT_1_R, resulting in reduced Na^+^ transport. We showed that NBCe2 is not affected by stimulation of the D_1_R or AT_1_R. Under basal conditions, NBCe1 is more active than NBCe2 (depicted as relatively larger directional transport arrows).

In an hRPTC containing the rs7571842 SLC4A5 SNP the model changes to what is depicted in **Fig 17**. Increasing intracellular Na^+^ concentration with high extracellular Na^+^ concentration or monensin (9 o’clock) increases NBCe2 mRNA, protein, and activity, while only marginally attenuating the protein and activity of NBCe1 (2 o’clock) in hRPTCs with SNPs in NBCe2. This results in a net increase in Na^+^ transport into the basolateral space. PAT1 activity (7 o’clock) increases because of an increase in intracellular bicarbonate (7 o’clock). NHE3 (9 o’clock) activity also increases because the increase in NBCe2 activity increases intracellular H^+^ following the conversion of transported HCO_3_^-^ to H_2_CO_3_ and its dissociation to H^+^ and HCO_3_^-^ resulting in a further increase in sodium reabsorption. An increase in HNF4A binding to the rs7571842 SLC4A5 increases its expression.

## Discussion

Increased renal sodium transport is involved in the pathogenesis of salt sensitivity with or without hypertension [1, 2, 9, 11–14, 23–29, 32–35, 37, 63, 68, 69]. The inability to eliminate the excess sodium intake can increase blood pressure in about 60% of hypertensive and approximately 25% of normotensive individuals, depending on racial background[68, 70]. The RPT regulates approximately 65% of renal sodium transport[71]. In the RPT, NHE3 is responsible for the majority of sodium transported across the RPT luminal membrane while Na^+^, K^+^/ATPase is responsible for the majority of sodium transported across the basolateral membrane. The sodium bicarbonate transporters, NBCe1 and NBCe2, are also involved in RPT sodium transport [7, 14, 17, 23, 29, 39, 71, 72].

The *SLC4A5* gene encodes for NBCe2 (aka NBC4). NBCe2 transports bicarbonate and sodium ions in a ratio of two-three to one, respectively [7, 73]. NBCe2 is expressed in the liver, heart, brain, and PT, mTAL, cTAL, and CD of the kidney [7, 16, 17, 20]. However, relatively low amounts of NBCe2 messenger RNA and protein have been found in the RPT in rats [16] and apical membrane of the RPT in humans on normal salt intake [17] and corroborated by the current report (Figs 1 and 2) in flash-frozen human kidney and primary cultures of RPTCs from fresh human kidneys, and immortalized hRPTCs. Our immunofluorescence studies demonstrating the presence of NBCe2 in hRPTC is corroborated by The Human Protein Atlas (www.proteinatlas.org). However, the minimal expression of NBCe2 in RPT and hRPTC in the basal state is increased by an increase in intracellular sodium [17] that was invariably observed in hRPTCs carrying *SLC4A5* HV, but less consistently in hRPTCs carrying *SLC4A5* WT. Extracellular apical membrane expression of NBCe2 has been demonstrated using TIRFM that places this protein in the top 70 nM of the membranes extracellular surface [17]. We now report that the presence of *SLC4A5* SNPs induces the binding of HNF4A to *SLC4A5*, resulting in an increase its transcription and hence the expression of NBCe2.

We used two methods to increase intracellular sodium to rule out nonspecific effects. We either increased extracellular sodium chloride or added monensin (10 μmol/L) which is used by many investigators to increase intracellular sodium[17, 50–57]. Monensin, at this concentration, has been reported to increase intracellular sodium by about 20 mmol/L in opossum kidney cells [57]. Since there was no difference in our results using extracellular sodium or monensin we ruled out the potential effect of monensin altering the trafficking of protein from the Golgi. Sodium chloride or sodium bicarbonate loading is associated with a decrease in RPT sodium transport via inhibition of NBCe1 and NHE3[22]. We found that when intracellular sodium was increased by monensin for 24 h, NBCe1 protein was decreased in hRPTCs carrying both WT and HV *SLC4A5*, but to a lesser degree in HV cells than in the WT hRPTCs, which could contribute to an attenuated suppression of bicarbonate transport in hRPTCs carrying *SLC4A5* (NBCe2) SNPs. This finding in WT hRPTCs is in agreement with the report showing that sodium loading of normotensive rats reduced the renal expression of NBCe1[22]. In WT hRPTCs, monensin changed the localization of NBCe1 from a membrane location to an intracellular location,[17] similar to that seen in monensin-treated parotid acinar cells[74]. As aforementioned, monensin is a non-selective ionophore, but used widely to increase intracellular sodium in various tissues[17, 50–57]. Therefore, we examined the effect of either monensin or high extracellular sodium in parallel in several experiments and found that either monensin or high extracellular sodium concentration increased NBCe2 protein in HV but not WT *SLC4A*5 RPTCs. The increase in intracellular sodium was also associated with a change in NBCe2 localization from highly compartmentalized perinuclear areas to diffuse punctate areas that extended to the luminal membrane shown in the current (**Fig 2K and 2L**) and a previous study[17]. This would be consistent with increased RPT sodium bicarbonate transport in hypertension [32–34].

*SLC4A5* variants rs10177833 and rs7571842 are associated with salt sensitivity in a Euro-Caucasian cohort of mixed sex[14], whereas *SLC4A5* variant rs8179526 is associated with salt resistance in African-American women [11] Therefore, we determined if the *SLC4A5* rs10177833 and rs7571842 cause loss or gain of function (i.e., sodium bicarbonate transport). We found that total (transcellular) sodium transport (from luminal membrane to outside the basolateral membrane) was increased in polarized hRPTCs with HV *SLC4A5* HV, relative to hRPTCs with WT *SLC4A5*. The increased total transcellular sodium flux in HV *SLC4A5* hRPTCs may be due to the increased expression and activity of NHE3 and NBCe2 at the luminal membrane, in spite of decreased expression and activity of NBCe1 as compared with WT cells. Indeed, the faster bicarbonate-dependent pH recovery in HV *SLC4A5* hRPTCs, induced by an increase in intracellular sodium due to monensin persisted even when SLC4A4 was silenced. By contrast, the faster bicarbonate-dependent pH recovery in HV SLC4A5 hRPTCs, induced by monensin was no longer evident when *SLC4A5* was silenced. Moreover, the increase in total sodium-dependent bicarbonate influx in polarized hRPTCs with *SLC4A5* variants also persisted, even when NHE3 activity was inhibited. Therefore, although increased activity of NHE3 is also associated with hypertension, the salt sensitivity associated with *SLC4A5* rs7571842 may indeed be due to a gain in its function. It is also possible that the increased activity of Cl^-^/HCO_3_^-^ [aka putative Cl^-^/HCO_3_^-^ anion transporter (PAT1)[62, 63, 75, 76] in hypertension may be linked to the increased activity of NBCe2. Hypertension in the metabolic syndrome has been suggested to be related to preserved RPT sodium bicarbonate cotransporter sensitivity to insulin[32, 34]. SS humans have enhanced renal tubular bicarbonate reabsorption[33]. Because NBCe1 function is decreased in RPTCs from SHR [23] the insulin-sensitive sodium bicarbonate cotransporter in the RPT in the metabolic syndrome and salt sensitivity could be NBCe2. In the current study, SS humans with *SLC4A5* HV had increased bicarbonate transport and faster pH recovery that could be attributed to NBCe2, because this observation persisted after silencing *SLC4A4*/NBCe1. Thus, we hypothesize that the SS phenotype could, at least in part, be related to the increased activity of NHE3, NBCe2, and PAT1 and the increased activity of NBCe2 may be secondary to an increase in its transcription.

An increase in renal bicarbonate transport in hypertension [32–34], however, is not consistent with the low serum bicarbonate and high anion gap in primary (essential) hypertension[21, 33, 77–80]. Primary hypertension is associated with increased acid production and the increase in renal bicarbonate transport may be secondary rather than primary. However, we propose that the increased expression of NBCe2 in the luminal membrane and decreased expression and activity of NBCe1 in the basolateral membrane of RPTs in salt sensitivity may be a mechanism that increases renal sodium transport when sodium intake is increased [14, 33, 77, 79, 80] and at the same time keeps bicarbonate transport into the blood stream lower than normal.

Dopamine, via D_1_-like receptors, can decrease sodium and bicarbonate transport in the RPT especially with a moderate sodium load [2, 25, 28, 29, 35, 37, 59, 60, 71, 72] or high tubular flow [38]. These effects are impaired in hypertension [2, 25, 28, 29, 35, 37, 59, 71, 72, 81]. In the current study, we found that stimulation of D_1_-like receptors had no effect on NBCe2 expression but enhanced the inhibitory effect of monensin on NBCe1 expression to a similar extent in hRPTCs carrying either WT or HV *SLC4A5*. This finding is in agreement with the increase in NBCe1 expression in mouse kidneys engineered to produce less RPT dopamine[82]. *SLC4A5* knockout mice are normotensive and do not become hypertensive until after an acid load. These mice are hypertensive presumably due to a compensatory increase in distal nephron sodium-bicarbonate transporters, e.g.*SLC4A7* [83] and ENaC [84]. Indeed, the elevated BP of *SLC4A5* knockout mice was normalized by bicarbonate treatment, presumably by preventing the activation of distal sodium transport[83]. Interestingly, an increase in bicarbonate consumption elevated BP in the WT mouse [83], some salt-sensitive and hypertensive humans have increased renal bicarbonate reabsorption [32–34].

Epigenetics is now appreciated to have an important role in the pathogenesis of cardiovascular disease, including hypertension[40, 85–89]. The Regulome database (www.regulomedb.org) contains a reference to HNF4A binding near a variant form of *SLC4A5*. In the kidney, HNF4A is present only in the RPT and no other segment of the nephron, and is involved with renal development[41, 42]. HNF4A DNA binding was increased in hRPTCs carrying HV *SLC4A5*. Furthermore, two HNF4A antagonists prevented the increase in NBCe2 activity in HV *SLC4A5* hRPTCs. Normally, on WT *SLC4A5* hRPTCs, the salt-induced increase in HNF4A expression may play a role in sodium homeostasis while also being an important regulator of many other metabolic pathways[41–47]. However, when *SLC4A5* variants rs10177833 and rs7571842 are present, the salt-induced increase in HNF4A expression takes on a new promiscuous role of increasing NBCe2 expression that leads to salt sensitivity. The fact that the SNPs are located in a non-promoter-related DNA intron suggests that they may act as an enhancer and that sodium-related binding may require additional cofactors to alter DNA structure or looping to a location remote to the binding event. However, determining the mechanism by which HNF4A increases expression of *SLC4A5* variants is beyond the scope of this report and will be the subject of future studies.

In conclusion, our results provide a possible mechanism on how increased NBCe2 activity associated with an attenuation of NBCe1 activity contributes to the salt sensitivity of BP. A model of the basal state of NBCe2 and NBCe1 is depicted in **Fig 16**. In HV *SLC4A5* hRPTCs the increase in NBCe2 activity is, at least in part, due to an increase in NBCe2 expression through a sodium-mediated increase in the interaction of HNF4A with *SLC4A5* variant rs7571842. The mechanism by which an increase in NBCe2 activity leads to an increase in sodium transport is depicted in the model in **Fig 17**. The finding of increased NBCe2 activity in hRPTCs from SS subjects has translational potential. We have developed a method to isolate and test living hRPTCs exfoliated into the urine and found that their response to monensin correlated with the clinically determined salt sensitivity of the subjects from which the hRPTCs were obtained[90]. Thus, we hope to develop this assay into a diagnostic test that will be capable of definitive and rapid determination of salt sensitivity without the cost and difficulty of administering a two week controlled diet [2].

## Supporting information

S1 Appendix Contains original data. (XLSX)

## Abbreviations

Ang II: angiotensin II
Ang III: angiotensin III
ChIP: chromatin immunoprecipitation
cTAL: cortical thick ascending limb
HV: homozygous variant
KD: knockdown
mTAL: medullary thick ascending limb
NHE3: sodium hydrogen exchanger type 3
OE: overexpressed
hRPTC: human renal proximal tubule cell
shRNA: short hairpin RNA
SR: salt-resistant
SS: salt-sensitive
VC: vector control
VEH: vehicle
WT: wild-type
HNF4A: hepatic nuclear factor 4 alpha
